# Elimination of African Onchocerciasis: Modeling the Impact of Increasing the Frequency of Ivermectin Mass Treatment

**DOI:** 10.1371/journal.pone.0115886

**Published:** 2014-12-29

**Authors:** Luc E. Coffeng, Wilma A. Stolk, Achim Hoerauf, Dik Habbema, Roel Bakker, Adrian D. Hopkins, Sake J. de Vlas

**Affiliations:** 1 Department of Public Health, Erasmus MC University Medical Center Rotterdam, P.O. box 2040, 3000 CA Rotterdam, The Netherlands; 2 Institute of Medical Microbiology, Immunology and Parasitology, University Hospital Bonn, Sigmund Freud Str. 25, 53105, Bonn, Germany; 3 Mectizan Donation Program, 325 Swanton Way, Decatur, Georgia, 30030, United States of America; University of Melbourne, Australia

## Abstract

The African Programme for Onchocerciasis Control (APOC) is currently shifting its focus from morbidity control to elimination of infection. To enhance the likelihood of elimination and speed up its achievement, programs may consider to increase the frequency of ivermectin mass treatment from annual to 6-monthly or even higher. In a computer simulation study, we examined the potential impact of increasing the mass treatment frequency for different settings. With the ONCHOSIM model, we simulated 92,610 scenarios pertaining to different assumptions about transmission conditions, history of mass treatment, the future mass treatment strategy, and ivermectin efficacy. Simulation results were used to determine the minimum remaining program duration and number of treatment rounds required to achieve 99% probability of elimination. Doubling the frequency of treatment from yearly to 6-monthly or 3-monthly was predicted to reduce remaining program duration by about 40% or 60%, respectively. These reductions come at a cost of additional treatment rounds, especially in case of 3-monthly mass treatment. Also, aforementioned reductions are highly dependent on maintained coverage, and could be completely nullified if coverage of mass treatment were to fall in the future. In low coverage settings, increasing treatment coverage is almost just as effective as increasing treatment frequency. We conclude that 6-monthly mass treatment may only be worth the effort in situations where annual treatment is expected to take a long time to achieve elimination in spite of good treatment coverage, e.g. because of unfavorable transmission conditions or because mass treatment started recently.

## Introduction

Since 1995, the African Programme for Onchocerciasis Control (APOC) has organized annual mass treatment with ivermectin in sixteen endemic African countries, with the aim to control eye and skin disease due to onchocerciasis [1]. Following the first reports of elimination of onchocerciasis from three savanna foci in West Africa with mass treatment alone and good progress towards this goal in two Nigerian foci [2]–[4], APOC has taken up the additional objective of eliminating infection, where possible [5]. To achieve elimination, it has been suggested that APOC should increase the frequency of mass treatment from annual to 6-monthly, following the example of the Onchocerciasis Elimination Program for the Americas (OEPA), which by means of 6-monthly and 3-monthly mass treatment has rapidly interrupted transmission in the majority of the American foci [6], [7]. Shorter and more intensive mass treatment programs are attractive as they speed up elimination, minimize the risk of interruption and emergence of drug resistance, which should be politically appealing to health officials [8]. However, increasing the frequency of mass treatment would also require major initial investments from APOC, endemic countries, and Merck, the pharmaceutical company donating ivermectin for onchocerciasis control. Also, the OEPA experience cannot be directly translated to the African setting due to differences in parasite-vector complexes (most Latin American vectors transmit onchocerciasis relatively inefficiently [9]) and program resources, scale, and implementation (OEPA: 500,000 people at risk of infection and vertically coordinated mass treatment [7]; APOC: 100 million at risk and community-directed mass treatment [1], [10]). Therefore, it is important to carefully evaluate where in Africa an increase in frequency is warranted, and where treatment should continue annually.

In most areas covered by APOC, annual mass treatment has been going on for 10 to 15 years at varying coverage levels [10], and it is not known how and to what extent the remaining program duration would change when switching to a higher mass treatment frequency. The consequences would vary between areas, depending on the local history of control in terms of duration and coverage of mass treatment in the past, and local transmission conditions such as pre-control infection level and inter-individual variation in exposure to fly bites [11]. Theoretically, higher treatment frequencies would be especially useful in reducing program duration (in absolute terms) in areas with high transmission rates and/or a short history of mass treatment. Empirical data on elimination of onchocerciasis from West African settings by means of 15–17 years of mass treatment are too sparse to inform a policy change regarding treatment frequency, as only in one of the reported areas (River Gambia focus) elimination was achieved by means of 6-monthly mass treatment [2], [4]. Furthermore, the 15–17 years of mass treatment may have been more than needed to achieve elimination. In absence of more empirical data, mathematical modeling may provide valuable insights.

In the current study, we investigated how increasing mass treatment frequency would affect the remaining program duration and the associated number of mass treatment rounds in African settings. We performed a computer simulation study with ONCHOSIM, a mathematical model for simulation of onchocerciasis transmission and control [12], [13]. This model has been previously used to predict the effects of onchocerciasis control in Africa [10], [11], [14]–[16]. In the current study, simulations were made based on various combinations of assumptions about transmission conditions; history of mass treatment; frequency, duration, and coverage of future mass treatment rounds; and the effects of ivermectin on adult male and female worms. In particular, we compared the effects of increasing frequency and increasing coverage of mass treatment, as the latter would probably require fewer investments and changes in ongoing programs.

## Methods

### The simulation model

ONCHOSIM is a micro-simulation model that simulates the life histories of persons and *Onchocerca volvulus* worms within persons. Simulated individuals are born and die, and are exposed to fly bites, which may transmit *O. volvulus* larvae from one person to another. ONCHOSIM simulates a closed population, meaning that there is no migration of potentially infected humans or flies into or out of the population. The probability that an individual is bitten by a fly is assumed to depend on age (between age zero and 20, exposure increases linearly from zero to a personal maximum), sex (women are assumed to experience 30% fewer fly bites than men), personal factors such as occupation and attractiveness to flies, and the season of the year. Transmitted larvae may develop into adult worms, which in turn produce new larvae or microfilariae (mf) when a person harbors at least one male and one female adult worm. The mf production of adult female worms is assumed to be zero during the worm's first year of life. After this pre-patent period, female worms are assumed to produce mf at maximum mf production capacity for five years, followed by a linear decline to zero over the course of 15 years (if a female worm lives that long). Adult worms are assumed to have an average reproductive lifespan (including the pre-patent period) of about 10 years, and 95% of worms are assumed to reach the end of their reproductive lifespan before the age of 13–14 years [17]. More information about quantification of demographic and biological parameters can be found in S1 Text.

The probability that a simulated individual participates in mass treatment with ivermectin is governed by age and sex (children under five years of age are not treated; a random proportion of women of reproductive age is not treated, assuming that they are pregnant or lactating), and a lifelong compliance factor (the higher the factor, the higher the probability that an individual participates in any given treatment round). Some individuals never participate in treatment, because they are chronically ill. More details about the model can be found in S1 Text.

### Assumptions about settings and future control scenarios

We simulated trends in infection levels for combinations of assumptions regarding settings (transmission conditions, history of mass treatment with ivermectin) and future mass treatment strategy and population coverage (Table 1). Assumptions were defined so as to be applicable to areas covered by APOC.

**Table 1:** Setting characteristics and treatment scenarios for simulations.

Settings and scenarios	Possible values
*Setting: transmission conditions*	
Seasonality	Year-round transmission^a^
Pre-control CMFL (community microfilarial load, the geometric mean microfilarial load in people of age 20 and above)	5, 10, 30, 55, 80 microfilariae per skin snip, corresponding to mf prevalence levels ranging from ∼45% to ∼85%, or 9,400 to 22,200 fly bites per adult male person per year^b^
Inter-individual variation in exposure to fly bites related to personal factors (e.g. attractiveness and occupation)	Low or high, specified as a gamma distribution for relative exposure to fly bites with mean value 1 and rate 3.5 (interquartile range 0.61–1.29) or 1.0 (IQR 0.29–1.39), respectively^c^
*Setting: history of control*	
Past mass treatment frequency	Annual
No. of mass treatment rounds provided until present	0, 1, 2, …, 14
Coverage in past mass treatment rounds (% of total population)	Coverage low (50%), intermediate (65%), or high (80%)
*Scenario: future mass treatment*	
Future mass treatment frequency	Annual, 6-monthly, or 3-monthly^d^
No. of future mass treatment rounds	0, 1, 2, …, 20, allowing estimation of the minimum number of future treatment rounds needed to achieve 99% probability of elimination
Coverage of future mass treatment rounds (% of total population)	Stable coverage (same as in the past), 15% lower^e^ (only for past coverage levels of 65% and 80%), or 15% higher^f^ (only for past coverage levels of 50% and 65%)

For each combination of the listed factors, we estimated the probability of elimination (zero prevalence of infection) 50 years after the last mass treatment, based on 1,000 repeated simulations in ONCHOSIM.

a Seasonality of fly biting rates was assumed to be proportional to the seasonal pattern observed in Asubende, Ghana [19]; the monthly biting rates (January–December) were assumed to be 104%, 91%, 58%, 75%, 75%, 66%, 102%, 133%, 117%, 128%, 146%, and 105% times the average monthly biting rate.

b CMFL values of 5 and 10 mf/ss are representative for APOC regions that were mesoendemic before the start of control (pre-control mf prevalence between 40% and 60%); the higher values of CMFL are representative for hyperendemic areas (mf prevalence>60%)[10]. We did not simulate areas with lower endemicity, where the expected duration of mass treatment is shorter.

c Low variation in exposure was combined with all possible values for pre-control CMFL. High variation in exposure was only combined with pre-control CMFL of 5 and 10 microfilariae per skin snip; assuming that for highly endemic areas individual variation in exposure to fly bites is not very high because of the multitude of flies (i.e. everyone is bitten very often).

d Simulated mass treatment rounds were scheduled on 1st of July (annual), just prior to the annual seasonal peak in fly biting rate, or additionally on the 1st of January (6-monthly treatment) and the 1st of April and 1st of October (3-monthly treatment). If the mass treatment program was assumed to switch from annual (past) to 6-monthly or 3-monthly (future) treatment, the future mass treatment scenario was scheduled to start on the 1st of January of the next year (i.e. six months after the last ‘past’ treatment), or on the 1st of October of the same year (i.e. three months after the last ‘past’ treatment), respectively.

e Hypothetically, increasing the frequency of mass treatment might induce treatment fatigue in the population, or lead people to think that it's not so bad to skip a mass treatment round as there will be another one in the near future.

f Hypothetically, increasing the frequency of mass treatment might increase awareness about onchocerciasis and motivate people to participate.

Transmission conditions were varied with regard to the average annual biting rate for adult male persons and the amount of inter-individual variation in exposure to fly bites due to personal factors (7 combinations in total). In general, higher inter-individual variation in exposure to fly bites leads to stronger overdispersion of infection [18], i.e. higher parasite concentrations in a few often bitten individuals, and lower parasite concentrations in all other, less often bitten individuals (S1 Fig.). The highly infected individuals contribute relatively more to transmission due to their high exposure to fly bites and their high skin mf densities, and therefore require more ivermectin treatments before they stop contributing to transmission of infection. Therefore, in settings with high variation in exposure to fly bites, elimination of infection requires longer program duration.

Seasonal variation in biting was assumed to always be proportional to seasonal patterns observed in Asubende, Ghana [19].

Past and future treatment strategies were defined in terms of number of rounds (315 combinations of 0 to 14 past treatment rounds and 0 to 20 future treatment rounds), mass treatment coverage in terms of the proportion of the whole population covered (7 combinations of maintained, decreasing, or increasing coverage), and frequency (3 options). Simulated mass treatment rounds were scheduled on 1^st^ of July (annual), just prior to the annual seasonal peak in fly biting rate, or additionally on the 1^st^ of January (6-monthly treatment) and the 1^st^ of April and 1^st^ of October (3-monthly treatment). If the mass treatment program was assumed to switch from annual (past) to 6-monthly or 3-monthly (future) treatment, the first of the “future” treatment takes place six or three months after the last “past” treatment round, respectively (i.e. on the 1^st^ of January of the next year or on the 1^st^ of October of the same year).

### Assumptions about ivermectin efficacy

Ivermectin was assumed to instantly kill all mf present in an individual. In addition, we assumed either of two alternative sets of assumptions about the effects of ivermectin on adult worms (Table 2). Assumption set 1 has also been used in previous simulation studies [10], [11], [15], and was quantified such that ONCHOSIM could reproduce trends in skin mf levels as observed in a community trial that encompassed five consecutive annual ivermectin treatments [14], [19]. Assumption set 2 was formulated to reflect evidence of the effects of ivermectin on adult worm survival and reproduction [20]–[30], and was quantified such that ONCHOSIM could reproduce trends in worm survival during three years of 3-monthly mass treatment, as estimated from nodulectomy data from Guatemala [23], and trends in skin mf levels up to two years after a single dose of ivermectin as reported in a published meta-analysis based on African and Latin American data [31] (see S1 Text for details). In both assumption sets, we assume that the effect of ivermectin on adult worms is independent of past treatment effects such that the effects of multiple treatments are multiplicative and such that there is no selection of drug-resistant worms.

**Table 2:** Two sets of assumptions about ivermectin efficacy in ONCHOSIM.

	Assumption set 1	Assumption set 2
Microfilaricidal effect	100%, instantaneous upon administration.	100%, instantaneous upon administration.
Macrofilaricidal effect	None.	Each treatment kills 6% of female adult worms and 12% of male adult worms.^a^ Pre-patent worms are not affected.
Temporary halt in production of microfilariae	All female worms temporarily stop producing mf. Production recovers gradually over time in all worms, reaching maximum production capacity after 11 months on average.^b^	Only female worms that were producing mf at the time of treatment temporarily stop producing mf. Production is resumed at full capacity after a random amount of time.^c^
Permanent reduction in adult female worm capacity to produce microfilariae	Average 35% reduction per treatment,^d^ with cumulative effects in worms repeatedly exposed to ivermectin	None.

Assumption set 1 was quantified such that ONCHOSIM could reproduce trends in skin mf levels as observed in a community trial that encompassed five consecutive annual ivermectin treatments [14], [19]. Assumption set 2 was quantified such that ONCHOSIM could reproduce trends in worm survival during three years of 3-monthly and 6-monthly mass treatment, as estimated from nodulectomy data [23], and trends in skin mf levels up to two years after a single dose of ivermectin as reported in a published meta-analysis [31]. Parameter values were fitted to the data with maximum likelihood, using the mean output of 100 repeated ONCHOSIM simulations as expected values (see **S1 Text** for details).

a Excess mortality has been reported for both female [23]–[30] and male worms [25], [26]. In the current study, excess mortality due to ivermectin was allowed to differ between male and female worms, reflecting the relative absence of male worms from subcutaneous nodules after repeated ivermectin treatment [22]–[30]. The macrofilaricidal effects of ivermectin were allowed to vary per treatment; however, this variation could not be estimated due to the aggregated nature of the Guatemalan data [23]. Instead, we arbitrarily assumed beta distributions with sample size 50 and mean 6% for males (2.5% and 97.5% percentiles 1.3%–14.0%) and 12% (3.9%–19.0%) for females, with the macrofilaricidal effects on male and female worms being perfectly correlated. Macrofilaricidal effects were assumed to be independent of earlier exposure to ivermectin and worm age, and hence reproductive capacity of the worm. In the sensitivity analysis, we set the average macrofilaricidal effects to either 4% and 8% (for males and females), or 9% and 18% (difference of factor 2/3 or 3/2) while keeping the sample size of the beta distribution at 50.

b This treatment effect was assumed to vary per worm and treatment; 2.5% and 97.5% percentiles 2–24 months.

c This assumption represents the notion that ivermectin causes temporary congestion of female worm uteri with dead mf, effectively preventing insemination and release of microfilariae [20], [21]. Time until recovery was assumed to vary per worm and treatment, and to follow an exponential distribution with mean 3.5 years (fitted to data [31]). This implies that 5% of adult female worms can be inseminated and release microfilariae within two months after exposure to ivermectin. Likewise, congestion resolves in 25%, 50%, 75%, and 95% of adult female worms within 1, 2.5, 5, and 10.5 years after exposure to ivermectin, respectively.

d To account for variation in treatment efficacy between persons and treatments, for every simulated person and treatment, the average reduction was multiplied with a random value drawn from a Weibull distribution with mean 1 and shape 2 (see also S1 Text). In the sensitivity analysis, the average reduction was set to 23% or 52% (difference of factor 2/3 or 3/2).

**Figure 1:**
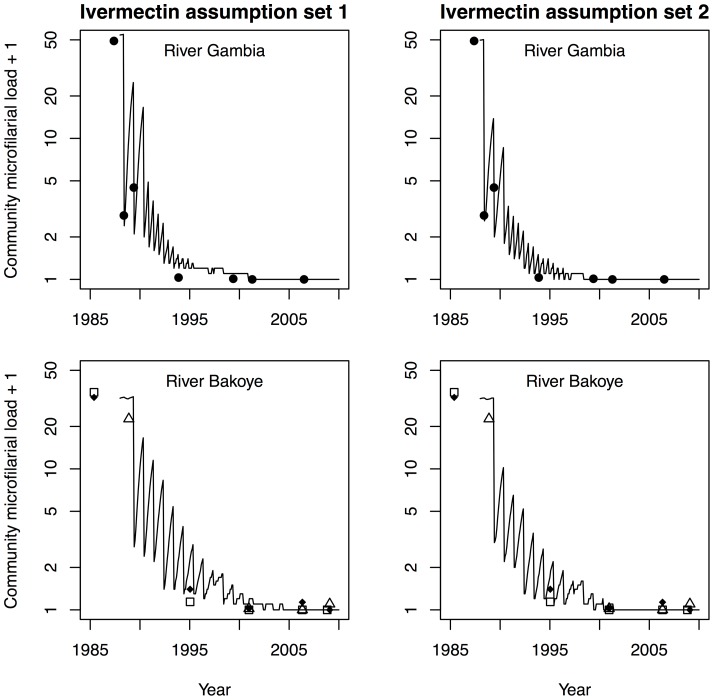
Comparison of ONCHOSIM-predicted trends in infection during 15 to 17 years of ivermectin mass treatment to previously published data. Data are from one hyperendemic village in the River Gambia focus in Senegal where annual and 6-monthly mass treatment took place (closed circles), and three hyperendemic village in the River Bakoye focus in Mali where only annual mass treatment took place (closed diamonds, open squares and triangles) [2], [4]. ONCHOSIM predictions (black lines) are the averages of 100 repeated simulations, which were based on either of two assumption sets for ivermectin efficacy (Table 2). After about ten mass treatment rounds (1994–1995), the model predictions based on ivermectin assumption set 1 are at most somewhat pessimistic compared to the data, though discrepancies may also be due to inaccuracy of data used to populate the model (e.g. information on pre-control infection levels and/or coverage and timing of mass treatment). The seemingly large discrepancies between predictions and data after the year 2005 are due to CMFL values close to zero that had been rounded down to one decimal before logarithmic transformation.

S2 Fig. illustrates example predictions for the effects of ivermectin on population infection levels, based on assumption sets 1 and 2, and three different mass treatment frequencies (annual, 6-monthly, and 3-monthly). Fig. 1 illustrates that model predictions based on either assumption set fit reasonably well to longitudinal data from hyperendemic villages along the Gambia and Bakoye River basins in West Africa, where 15 to 17 years of annual ivermectin mass treatment (River Gambia focus: 6-monthly from 1990 onwards) have led to elimination of onchocerciasis [2], [4]. Predictions for mesoendemic and hypoendemic villages in the same areas fitted the data at least as well as predictions for hyperendemic areas.

### Sensitivity analysis

In the sensitivity analysis, we investigated the impact of changing assumptions about the magnitude of negative density dependence in transmission and the permanent effects of ivermectin on adult worms. Here, negative density dependence refers to the situation where transmission of infection becomes relatively more efficient at lower levels of infection; stronger negative density dependence means a stronger increase in transmission efficiency. In our baseline simulations we used the default quantification of biological key parameters for savanna type of infection, based on data collected by the Onchocerciasis Control Programme in West Africa, assuming strong negative density dependence in transmission. However, for forest areas it has been suggested that negative density dependence in transmission is less pronounced [32]. To reflect this in the sensitivity analysis, we assumed a less concave pattern in saturation of transmission (i.e. at low densities of skin mf, uptake of skin mf by *Simulium* flies is more linearly associated with skin mf density; see footnotes on page 6 of S1 Text for exact quantification).

Because parameters for ivermectin efficacy were based on a limited number of datasets that cover two to five years, we also varied our assumptions about the permanent effects of ivermectin on adult worms. For assumption set 1 regarding ivermectin efficacy, we assumed that the permanent reduction in worm capacity to produce mf was a factor 2/3 lower or 3/2 higher than in the main analysis (Table 2). Likewise, for assumption set 2 we assumed that the macrofilaricidal effects of ivermectin were a factor 2/3 lower or 3/2 higher.

### Simulations

Because many processes simulated in ONCHOSIM involve probabilities, repeated model simulations based on the same assumptions will results in slightly different predictions because of stochastic variation. We estimated the probability of elimination, based on the fraction of 1,000 repeated simulations that result in elimination. Elimination was defined as absence of infection 50 years after the last mass treatment, where infection diagnosis was based on two skin snips per person (assuming that the chance of finding zero mf-positive individuals among all simulated individuals (∼400) is negligible during sustainable transmission). The rationale for evaluating the occurrence of elimination 50 years after the last treatment round, and not after a shorter period of say 1–5 years, is as follows. As laid out by the breakpoint theory [33], the prevalence of infection does not need to be reduced to zero by the time that mass treatment stops. Below some threshold (the ‘breakpoint’), the probability that a worm successfully reproduces and brings forth at least one new reproducing worm falls below 1 so that the worm population will gradually extinct. The period of 50 year is expected to be long enough to allow for this natural extinction. Probability of elimination was thus estimated for each combination of assumptions (92,610 in total), assuming a hypothetical village with 400 inhabitants (a village size typical for rural Africa). For every combination of transmission setting, history of control, future control strategy, and ivermectin efficacy (4,410 combinations), we determined the minimum number of future treatment rounds required to achieve ≥99% probability of elimination, if possible within the simulated range of 0–20 future treatment rounds. (The exact binomial 95%-confidence interval for 99% probability of elimination based on 1,000 simulations is 98.1%–99.5%.) The associated remaining program duration was calculated by dividing the number of future treatment rounds by the future treatment frequency per year.

Simulations were performed on the Dutch Life Science grid (http://www.surfsara.nl/project/life-science-grid), a UNIX-based computer grid network shared between several Dutch universities and academic institutes. Simulation results were processed in R (version 2.13.2).

### Calculating relative changes in program duration and number of mass treatment rounds

To summarize the impact of a change in mass treatment frequency, we estimated the relative change in remaining program duration and remaining number of treatment rounds, using the scenario of continuing mass treatment annually at maintained coverage as a reference. We pooled estimates over the different scenarios for the number of past treatment rounds (relative reductions were very similar for different numbers of past treatment rounds), and for a high-level overview over different scenarios for pre-control infection levels as well (assuming that these different scenarios are equally likely to occur). Pooled estimates of the relative change were calculated as 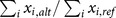, where *x* is the remaining program duration or number of treatment rounds until elimination under the reference (*ref*) and alternative scenarios (*alt*; defined in terms of coverage and frequency of mass treatment), and *i* represents different scenarios for the number of past treatment rounds and optionally, different pre-control infection levels. We did not pool over different assumptions regarding variation in exposure to fly bites or ivermectin efficacy.

## Results

**Figure 2:**
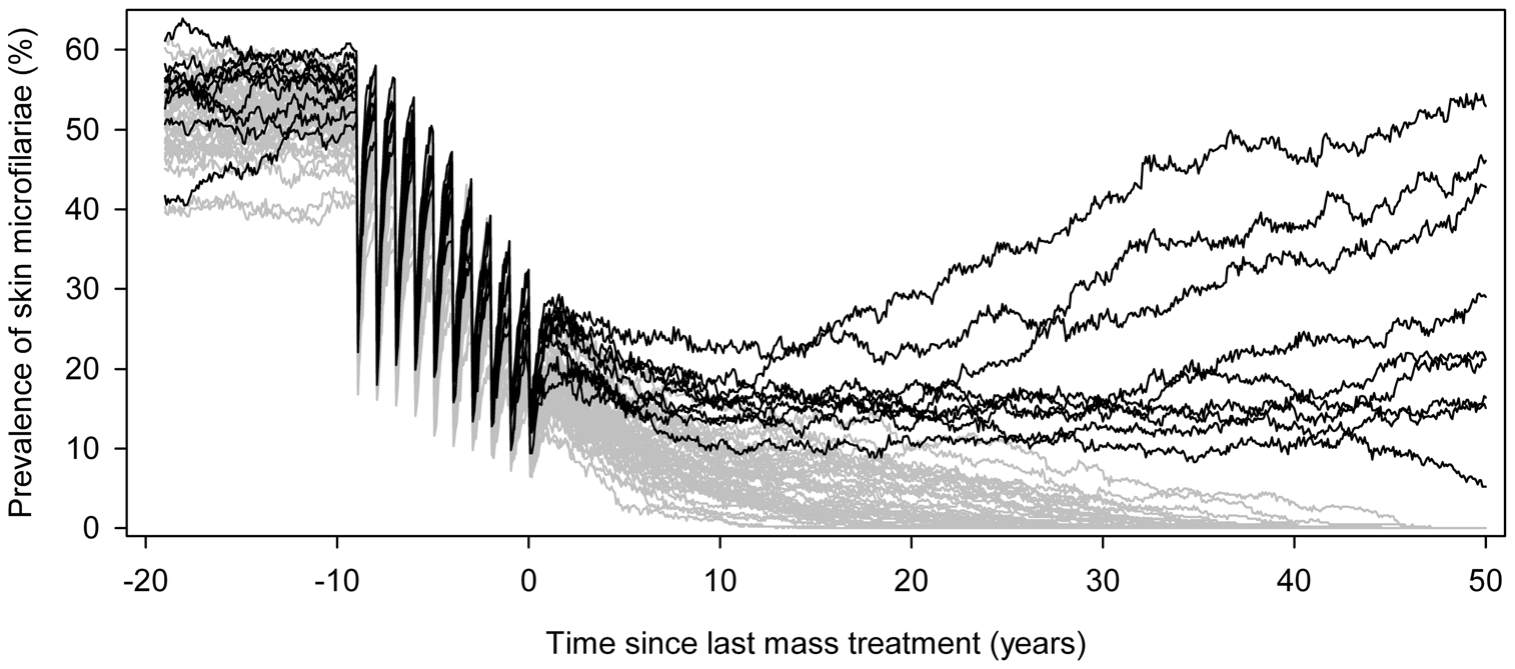
Example prediction for the prospect of elimination, generated by ONCHOSIM. The graph shows expected trends for a setting with 10 past annual treatment rounds with 65% population coverage, if treatment is not continued into the future. Time 0 represents the current situation and the last treatment was given just before time 0. We assumed a pre-control community microfilarial load of about 10 mf per skin snip, which is equivalent to a crude prevalence of skin microfilariae of about 50%; low variation between individuals in relative exposure to fly bites; and ivermectin efficacy according to assumption set 1 (Table 2). Each of the 50 lines represents a single simulation of a typical rural village population in Africa (about 400 individuals). Graph line colors indicate whether a simulation contained individuals with detectable skin microfilariae 50 years after the last mass treatment (black lines, n = 9), or not (grey lines, n = 41). In this example, the probability of elimination would be estimated at 41/50 = 82%. The erratic appearance of the graph lines is due to the stochastic nature of the simulations.

Fig. 2 shows ONCHOSIM-predicted trends in prevalence of skin microfilariae (mf prevalence) and illustrates how the probability of elimination is estimated. This figure represents a setting where 10 annual treatment rounds took place in the past (before time 0) and treatment is not continued into the future. To achieve elimination, it is not necessary to clear all infection before mass treatment can be stopped. In this case, 10%–30% of the population is still mf positive after 10 rounds. However, the mean infection intensity is reduced to very low levels: past treatments have reduced skin mf densities and the number of new worm that is introduced into the human population. Remaining worms have reduced mf productivity due to treatment effects and relatively old age. In combination with the moderate biting rate that occurs in this mesoendemic setting, this explains why the incidence of new infections is low and that there is a considerable chance of elimination: 41/50 simulations resulted in elimination, yielding an estimated 82% probability of elimination (exact 95%-confidence interval: 69%–91%). Note that in highly endemic settings with higher biting rates, the mf prevalence and intensity will have to be reduced to lower levels to reach the same probability of elimination, and more treatment rounds will be required to achieve this.

**Figure 3:**
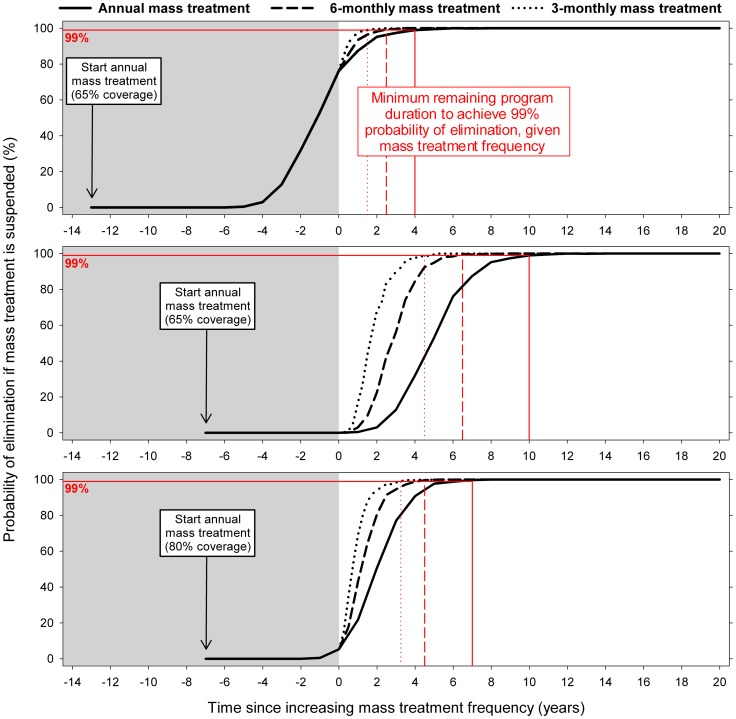
Predicted trends in probability of elimination over time for settings with different history of control. The three panels represent predictions for different histories of control in terms of number of past treatment rounds (14 or 8) and mass treatment coverage (65% or 80%). Black lines represent the probability of elimination (y-axis) if mass treatment were to be suspended at a certain point in time (x-axis). Trends until now (time 0) are displayed against a shaded background, while expected future trends are shown against a white background. Different line types pertain to different future mass treatment frequencies (annual, 6-monthly, or 3-monthly). Red lines highlight the predicted minimum remaining program duration required to achieve 99% probability of elimination (based on 1,000 repeated simulations). The three panels are equal with respect to assumed transmission conditions (pre-control community microfilarial load of about 30 mf per skin snip, low variation between individuals in relative exposure to fly bites) and ivermectin efficacy (assumption set 1). Elimination was defined as absence of infection 50 years after suspension of mass treatment.

Fig. 3 illustrates how the probability of elimination (y-axis) increases with program duration (x-axis); the program stops when the elimination probability reaches 99% (red drop-down lines). This figure shows how increasing mass treatment frequency from now on (time  = 0) would reduce the remaining program duration required. In the top panel, for example, a shift from annual to 6-monthly treatment would reduce the remaining program duration from 4 years to 2.5 years (37.5% reduction). This reduction is associated with an increase in the remaining number of mass treatment rounds from 4 to 5 (2 rounds per year x 2.5 years; 25% increase). In general, the longer the expected remaining program duration under annual treatment, the larger the absolute reduction achieved by shifting from annual treatment to higher frequency treatment. Thus, the impact of increasing mass treatment frequency on remaining program duration in absolute terms was larger if mass treatment started more recently (Fig. 3, middle vs. top panel) and when mass treatment coverage was lower (middle vs. bottom panel). Similarly, the time reduction would be larger in settings with more unfavorable transmission conditions (e.g. high pre-control infection levels and/or high variation in exposure to fly bites).

**Figure 4:**
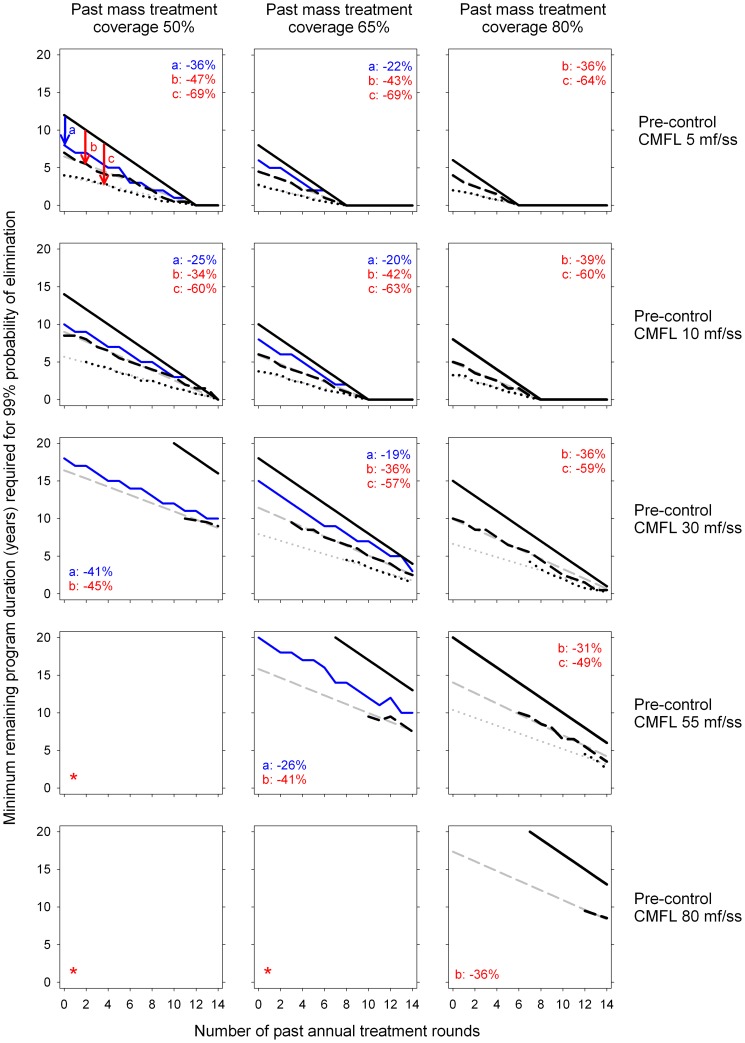
Predicted minimum remaining program duration required until elimination of onchocerciasis, assuming ivermectin efficacy as in assumption set 1 and low inter-individual variation in exposure to fly bites. Panels illustrate the minimum remaining program duration (y-axis) required for 99% probability of elimination (absence of infection 50 years after the mass last treatment), given the number of annual mass treatment rounds already completed (x-axis), as predicted by ONCHOSIM (1,000 simulations per scenario). Each panel compares four strategies: continuing annual mass treatment at same coverage (solid black line), switching to 6-monthly mass treatment at same coverage (dashed black line), switching to 3-monthly mass treatment at same coverage (dotted black line), or continuing annual treatment at increased coverage (+15 percentage points; solid blue line; only for past mass treatment coverage of 50% and 65%). Different panels pertain to increasing pre-control infection levels (top to bottom), and increasing values of past mass treatment coverage (left to right). Grey lines represent smoothed and where relevant extrapolated trendline of simulated outcomes, fitted such that they intersect with the x-axis at the same point as graph lines for annual mass treatment (black solid lines). Values in the corner of each panel represent reductions in remaining program duration (pooled over scenarios for different numbers of past treatment rounds), when increasing coverage (a), switching to 6-monthly mass treatment (b), or switching to 3-monthly mass treatment (c), compared to continuing annual treatment at the same coverage. Panels marked with an asterisk (*) pertain to simulations that did not result in 99% probability of elimination within 20 future treatment rounds, and hence contain no graph lines.

Fig. 4 shows the minimum remaining program duration required for 99% probability of elimination (y-axis), in relation to the number of (annual) mass treatment rounds already completed (x-axis), and the future mass treatment strategy. Shifting from annual to 6-monthly treatment reduces the remaining program duration by about 40%, and this is more or less independent of setting characteristics (number of treatment rounds already provided, average coverage, pre-control endemicity level). The reduction is always less than 50%, implying that the number of treatment rounds always increases. The figure also shows the effect of increasing treatment coverage instead of frequency (blue lines). Increasing coverage of annual mass treatment from 50% to 65% causes a reduction in the remaining program duration, similar in magnitude to the reduction achieved by increasing the frequency to 6-monthly (left column of panels). In contrast to increasing frequency, increasing coverage also causes a reduction in the remaining number of treatment rounds. Further increasing coverage from 65% to 80% has a relatively smaller impact on remaining duration (middle column of panels). Predictions based on ivermectin efficacy as in assumption set 2 were similar to those based on assumption set 1 (S3 Fig.). Only predictions for annual treatment were slightly more optimistic (1–2 years shorter program duration) when based on assumption set 2. As expected, high inter-individual variation in exposure to fly bites was associated with longer program duration required for elimination (S4 and S5 Figs.). Still, the relative differences between future mass treatment strategies were similar to those based on the assumption of low inter-individual variation in exposure to fly bites.

Table 3 shows the relative change in remaining program duration resulting from a change in treatment frequency or treatment coverage. The table also shows the associated change in remaining number of treatment rounds. Reductions in program duration were highest for situations where past mass treatment coverage was relatively low, and vice versa. Whereas increasing coverage only was always associated with a reduction in the number of mass treatment rounds, switching to high frequency mass treatment was associated with an increase in number of mass treatment rounds, except when coinciding with an increase in coverage. Further, if switching to high frequency mass treatment would coincide with a drop in coverage of 15 percentage points (e.g. due to perceived lower importance of participation among the target population), this strongly attenuated (and sometimes even completely nullified) the reduction in the program duration, and would lead to a further increase in the number of future treatment rounds required. All aforementioned patterns were more pessimistic for predictions based on the assumption of ivermectin efficacy according to assumption set 2 (lower reduction in program duration, higher increase in number of treatment rounds; rightmost two columns of Table 3).

**Table 3:** Effects of future control strategy on remaining program duration and treatment rounds until elimination.

Mass treatment strategy			Ivermectin assumption set 1		Ivermectin assumption set 2	
Past coverage	Future coverage	Future frequency	Program duration	Number of treatment rounds	Program duration	Number of treatment rounds
50%	50%	6-monthly*	−41%	+18%	−34%	+32%
		3-monthly*	−64%	+43%	−58%	+69%
	65%	annually*	−37%	−37%	−32%	−32%
		6-monthly*	−60%	−21%	−36%	+28%
		3-monthly*	−75%	0%	−77%	−7%
65%	50%	6-monthly**	−14%	+72%	−3%	+95%
		3-monthly**	−46%	+118%	−32%	+151%
	65%	6-monthly*	−40%	+21%	−29%	+42%
		3-monthly*	−62%	+51%	−52%	+92%
	80%	annually*	−23%	−23%	−17%	−17%
		6-monthly*	−48%	+4%	−12%	+76%
		3-monthly*	−69%	+26%	−69%	+23%
80%	65%	6-monthly**	−25%	+50%	−15%	+69%
		3-monthly**	−52%	+91%	−46%	+118%
	80%	6-monthly*	−35%	+31%	−26%	+48%
		3-monthly*	−59%	+66%	−49%	+103%

All differences are defined compared to the strategy of continuing annual treatment strategy at maintained treatment coverage, and are based on the assumptions of low variation in exposure to fly bites. Estimates are pooled over all combinations of number of past treatment rounds and pre-control community microfilarial load (see Figs. 4, S3, S4, and S5 for more detailed estimates of reduction in program duration by pre-control community microfilarial load).

* Estimates were similar for different assumptions about pre-control levels of infection and number of past treatment rounds.

** When future mass treatment coverage was assumed to drop, the reduction in program duration tended to be smaller for settings with fewer past treatment rounds and higher pre-control infection levels (and vice versa). Analogously, when future mass treatment coverage was assumed to drop, the increase in remaining number of mass treatment rounds tended to be higher for settings with fewer past treatment rounds and higher pre-control infection levels (and vice versa).

Table 4 summarizes the findings from the sensitivity analysis. If negative density dependence in transmission was assumed to be less pronounced than in the main analysis, program duration required to achieve 99% probability of elimination was shorter. However, the relative difference in program duration between continuing annual mass treatment or switching the 6-monthly mass treatment remained approximately the same. If the permanent effects of ivermectin on adult worms were assumed to be larger, program duration required for elimination was shorter, but increasing coverage or the frequency of mass treatment added relatively less to speeding up elimination (as annual mass treatment was already very effective). Conversely, assuming smaller permanent effects of ivermectin resulted in longer program duration and relatively more benefit from increasing coverage or frequency of mass treatment.

**Table 4:** Sensitivity analysis for assumptions about density dependence in transmission and permanent effects of ivermectin on adult worms.

Assumption set regarding ivermectin efficacy	Number of past treatment rounds at 65% coverage	Analysis	Minimum number of treatments and program duration required to achieve 99% probability of elimination, given future mass treatment strategy*			
			Annual treatment at 65% coverage	6-monthly treatment at 65% coverage		Annual treatment at 80% coverage
				Number of treatment rounds	Program duration	
1	0	Main analysis	18	>20**	>10**	15 (−17%)
		Lower density dependence	13	17 (+31%)	8.5 (−35%)	10 (−23%)
		Smaller reduction in worm fertility***	>20**	>20**	>10**	18
		Larger reduction in worm fertility***	15	>20**	>10**	12 (−20%)
	6	Main analysis	12	15 (+25%)	7.5 (−38%)	9 (−25%)
		Lower density dependence	7	9 (+29%)	4.5 (−36%)	6 (−14%)
		Smaller reduction in worm fertility***	17	19 (+12%)	9.5 (−44%)	12 (−29%)
		Larger reduction in worm fertility***	9	13 (+44%)	6.5 (−28%)	7 (−22%)
2	0	Main analysis	18	>20**	>10**	15 (−17%)
		Lower density dependence	13	19 (+46%)	9.5 (−27%)	11 (−15%)
		Smaller macrofilaricidal effects***	20	>20**	>10**	16 (−20%)
		Larger macrofilaricidal effects***	17	>20**	>10**	13 (−24%)
	6	Main analysis	12	16 (+33%)	8 (−33%)	10 (−17%)
		Lower density dependence	7	10 (+43%)	5 (−29%)	6 (−14%)
		Smaller macrofilaricidal effects***	14	18 (+29%)	9 (−36%)	11 (−21%)
		Larger macrofilaricidal effects***	11	14 (+27%)	7 (−36%)	9 (−18%)

The results presented here are based on a setting where the pre-control community microfilarial load is 30 microfilariae per skin snip.

* Numbers in parentheses represent differences relative to the strategy of continuing mass treatment annually at 65% coverage.

** The probability of elimination was less than 99% within the scope of the simulations (maximum 20 future treatment rounds).

*** Permanent effects of ivermectin on adult worms were assumed to be either a factor 2/3 lower or a factor 3/2 higher (see Table 2 for details).

## Discussion

With the mathematical simulation model ONCHOSIM, we predicted how a shift from annual to 6-monthly or 3-monthly ivermectin mass treatments changes remaining program duration and number of mass treatment rounds required for 99% probability of elimination. We predicted that high frequency mass treatment at maintained coverage will reduce duration until elimination by as much as 40% (6-monthly mass treatment) or 64% (3-monthly mass treatment), though always at a cost of additional treatment rounds. In low coverage settings, reductions in remaining program duration can be achieved just as well by increasing treatment coverage as by increasing treatment frequency to 6-monthly. Further, while an increase in both frequency and coverage of mass treatment would work synergistically and could in some settings even decrease the number of mass treatment rounds required for elimination, a drop in coverage could strongly attenuate or even completely nullify the reduction in program duration, especially in areas with high levels of residual infection and high potential for transmission.

### Ivermectin efficacy and effectiveness

Our results were generated using computer simulation, as empirical evidence from past and ongoing programs in Africa based solely on ivermectin mass treatment is still limited and difficult to generalize [2]–[4]. An important uncertainty concerns the efficacy of ivermectin treatment on adult worms and especially the effects of repeated treatments. Some argue that the macrofilaricidal effect of ivermectin may be enhanced with high frequency treatment [22], [26], [27]; others debate whether or not the effects of multiple treatments are cumulative [34], [35]. Turner *et al*. [36] showed that the estimated duration of mass treatment strongly depends on whether or not multiple treatments were assumed to have cumulative effects, but made no attempt to validate the treatment efficacy assumptions by fitting the model to data. The two assumption sets about ivermectin efficacy used in this study (Table 2) both resulted in a plausible reproduction of trends of infection observed in West Africa (Fig. 1), suggesting that there is a cumulative effect on net mf production, either through an effect on mf productivity or through a macrofilaricidal effect. More detailed data would be required to tease out the partial contributions of the macrofilaricidal and embryostatic effects of ivermectin. Although uncertainty remains regarding the exact mechanisms, this uncertainty does not seem to affect the conclusions from this studies: the required duration of mass treatment in different settings was very similar for the two sets of assumptions, as was the impact of changing frequency on the required future duration. Also related to uncertainty about ivermectin efficacy, in the sensitivity analysis we show how high frequency mass treatment has less added benefit if treatments by themselves have greater permanent effects on adult worms, and vice versa. With regard to the actual effectiveness of ivermectin mass treatment, patterns of coverage and systematic non-compliance are another source of uncertainty, and a cause of variation between locations.

### Drug resistance

In our simulation exercise, we assumed that there is no selection of drug-resistant parasites. However, there have been concerns about suboptimal responders (which we do take account of in our simulations), as reported in Ghana [37], occurring because of possible emergence of drug resistance [38], [39]. More recently, it has been reported that parasitological responses to ivermectin treatment are possibly less favorable in multiply-treated populations than in treatment-naive populations, both in terms of skin mf dynamics [35] and worm reproductive status [40]. The emergence of (partly) resistant parasites can endanger prospects of elimination. Although our study does not provide an answer, theoretically, switching to high frequency mass treatment may help eliminate infection before resistance becomes uncontrollable. Additional modeling is required to address this uncertainty.

### Geographical variation in transmission

ONCHOSIM is currently parameterized and calibrated to simulate year-round transmission in savanna areas, whereas a large part of the APOC region is covered by forest with other parasite-vector-complexes [10]. In particular, density dependence in transmission and inter-individual variation in exposure to fly bites may differ between forest and savanna areas [9], [32], [41]–[43]. In ONCHOSIM, density dependence in transmission is assumed to be negative (i.e. transmission becomes less effective at high levels of infection) and is entirely governed by a saturating association between mf uptake by the vector and skin mf loads in the host. There is evidence suggesting that negative density-dependence in mf uptake is less pronounced in forest areas than in savanna areas [32]. However, it is unclear to what extent other density-dependent mechanisms in the host and/or fly make up for this [9]. In the sensitivity analysis where we assumed less pronounced negative density dependence in mf uptake, we found that the reduction in program duration by switching to 6-monthly treatment was similar to that in the main analysis. With regard to inter-individual variation in exposure to fly bites, our simulations suggest that reductions in program duration by high frequency mass treatment are similar for situations with high and low variation. Further, in areas where transmission is highly seasonal and mass treatment is already taking place right before the transmission season, the additional impact of 6-monthly or 3-monthly treatment is expected to be lower than we predict here, as at the time of the second mass treatment in a year, transmission rates are already low. Related to this, in areas with highly seasonal transmission, optimizing the timing of mass treatment (if not already right before the seasonal peak in fly biting rates) may also help to reduce program duration without major new investments (similar to increasing treatment coverage). More simulations are needed to provide insight into the magnitude of this effect. Last, because probability of elimination is in principle lower in more highly populated communities (due to lower probability of extinction of infection by chance), it is important to note that our predictions are for communities of about 400 individuals. I.e., smaller communities may require fewer mass treatment rounds to achieve elimination than predicted here (assuming all else equal), and vice versa.

### Migration of humans and flies

In our simulations, we assumed no migration of infected flies or humans. This assumption is not problematic if humans and flies originate from areas with a similar history of control. However, our predictions for the program duration required for elimination do not hold for areas that are subject to immigration of high numbers of infected humans and/or flies from other areas, e.g. projects bordering areas where transmission is ongoing at a relative high rate (potentially between mass treatment rounds) because control started much later or has not yet started (e.g. due to civil unrest or *Loa loa* coendemicity), or savanna areas that experience migration of infected flies from further away (savanna-type flies may travel long distances on the wind). Therefore, it is important that transmission zones are identified and that areas and countries within such zones continue to coordinate their control programs, to prevent migration from delaying elimination [5], [44].

### Comparison to a previous simulation study

The effects of frequency and coverage of mass treatment on prospects of elimination have been previously studied with ONCHOSIM by Winnen *et al*. [11]. However, the current study considers more settings and scenarios, and accounts for the fact that in large parts of Africa, onchocerciasis control has been ongoing for some time. Further, the current study provides more precise estimates of the prospects of elimination, based on 1,000 simulations for each of 92,610 scenarios. Winnen *et al*. concluded that duration until elimination of a program based entirely on 6-monthly mass treatment would be less than half that of a program based entirely on annual treatment. In light of our predictions, this now seems too optimistic. The more optimistic estimates from the study by Winnen *et al*. result from a suboptimal design of the simulation experiment: Winnen *et al*. performed 30.000 simulation runs, each based on a different set of random values for relevant ONCHOSIM input parameter, and they analyzed prospects of elimination (a binary outcome) as a function of the aforementioned parameter values by means of logistic regression. Estimates of the number of treatment rounds required to achieve 99% probability of elimination under different conditions and of the reduction resulting from an increase in treatment frequency, are based on a relatively few simulation runs and sensitive to the distributions of parameter values from which the random values are generated (as we found out by reproducing the entire Winnen study with the new JAVA-based ONCHOSIM). The current study avoids this pitfall by using many repeated simulations per scenario and avoiding regression modeling.

### Cost and benefit

Compared with annual mass treatment, high frequency mass treatment is more resource demanding, due to costs related to its implementation, logistics, and the higher total number of mass treatment rounds required to achieve elimination. In light of on-going discussions on how to improve onchocerciasis control strategies [6], [7] it may seem attractive to increase mass treatment frequency in poorly performing projects. However, according to our simulations, in low coverage settings (coverage around 50% or lower), increasing coverage by 15% or more can be just as effective as increasing frequency of mass treatment, while being less resource-demanding, requiring fewer mass treatment rounds, and most likely, fewer investments in the supply chain (e.g. drug transport and storage capacity). Switching to 6-monthly mass treatment may only be worth the effort in situations where annual treatment is expected to take a long time to achieve elimination in spite of good treatment coverage, e.g. because of unfavorable transmission conditions or because mass treatment started recently. Increasing the frequency to 3-monthly seems very unattractive as it may lead to doubling of the number of treatment rounds required for elimination. In contrast, increasing both coverage and frequency, if feasible, may even reduce the remaining number of mass treatment rounds required until elimination. Whether this results in cost savings depends entirely on the investments required for implementing alternative future mass treatment strategies, and the additional cost of maintaining them. Turner *et al*. recently estimated that in Ghana, the cost of 6-monthly mass treatment against onchocerciasis was about 50–60% higher than the cost of annual mass treatment (excluding cost of donated drugs) [45] which is comparable to the additional cost of 6-monthly over annual mass treatment against lymphatic filariasis [8]. However, these additional costs most likely would vary geographically, as they will depend on road conditions, spread of communities, population densities, compensation systems for volunteers responsible for local drug distribution, and potential integration of interventions against different diseases [36].

### Requirements for implementation of high frequency mass treatment

There are several requirements and barriers for the implementation of high frequency mass treatment. First, adequate planning is required to guarantee sufficient drug supplies. Second, communities targeted for high frequency mass treatment would have to be sensitized, as in Africa ivermectin mass treatment has been implemented using a community-directed approach [1]. Community sensitization is needed to convince people that it is in their individual and community interest to participate in each treatment so that treatment coverage does not drop (which could nullify the potential impact of high frequency mass treatment). Third, high frequency mass treatment would have to be harmonized with ongoing integrated control of onchocerciasis and other tropical diseases [46], [47]. Fourth, implementation of high frequency mass treatment may be difficult because of heavily burdened countries' health systems [48], and community volunteers. For instance, in Ghana, it has been reported that increasing mass treatment frequency was associated with disease control officers spending substantially more time on reporting activities [45]. Fifth, from the healthcare provider's point of view, implementation of high frequency mass treatment may require an estimated 50–60% increase in average annual program cost [45]. Whether this is counterbalanced by a decrease in total program costs, as has been predicted for switching from annual to 6-monthly treatment for elimination of lymphatic filariasis [8], will depend on the magnitude of potential efficiency gains and the absolute reduction in program duration. Last, high frequency mass treatment may not be feasible everywhere due to weather, seasonal migration of populations, logistical considerations.

### Choosing a mass treatment strategy

For planning and advocacy purposes, national onchocerciasis elimination programs will most likely be required to achieve elimination within a certain timeframe, as is already the case for several other neglected tropical diseases [49]. Our predictions provide information on what is reasonable to expect in terms of time until elimination when a certain mass treatment strategy is successfully implemented (S1 File). This information can help set reasonable timelines, taking into account the current program performance (is coverage at least 65%?) and the specific situations of such countries (which strategy is feasible?). Further, our prediction provide information that will allow elimination programs to estimate the approximate amount of resources required for achieving elimination, and choose which strategy will most likely get the job done with the minimum amount of resources.

### Conclusion

In Africa, shifting to 6-monthly mass treatment with ivermectin will shorten the program duration required for onchocerciasis elimination. The associated increase in the remaining number of mass treatment rounds is probably worth the effort in settings where annual treatment may be expected to still take a long time to achieve elimination in spite of good coverage, e.g. because of unfavorable transmission conditions or because mass treatment started recently. In low coverage settings, priority should be given to increasing mass treatment coverage, as this is a less resource-demanding option that is similarly effective. The benefits of increasing mass treatment frequency will be highly dependent on maintained high coverage, and could be completely nullified if coverage were to fall after increasing mass treatment frequency.

## Supporting Information

S1 Fig **Pre-control skin microfilarial density distribution for the seven transmission settings.**
(PDF)

S2 Fig **ONCHOSIM predictions for community infection levels, based on two sets of assumptions about ivermectin efficacy.** Ivermectin was assumed to instantly kill all mf present in an individual. In addition, we assumed either of two alternative sets of assumptions about the effects of ivermectin on adult worms (left and right panels; for details see Table 3). The frequency of ivermectin mass treatment was assumed to be either annual (solid lines), 6-monthly (dashed lines), or 3-monthly (dotted lines). The trends depicted here are the averages of 100 simulations of a hypothetical village with 400 inhabitants and a pre-control community microfilarial load of 30 microfilariae per skin snip. Ivermectin mass treatment was assumed to cover 65% of the population (∼80% of eligible population).
(PDF)

S3 Fig **Predicted minimum remaining program duration required until elimination of onchocerciasis, assuming ivermectin efficacy as in assumption set 2 and low inter-individual variation in exposure to fly bites.** Panels illustrate the minimum remaining program duration (y-axis) required for 99% probability of elimination (absence of infection 50 years after the last mass treatment), given the number of annual mass treatment rounds already completed (x-axis), as predicted by ONCHOSIM (1,000 simulations per scenario). Each panel compares four strategies: continuing annual mass treatment at same coverage (solid black line), switching to 6-monthly mass treatment at same coverage (dashed black line), switching to 3-monthly mass treatment at same coverage (dotted black line), or continuing annual treatment at increased coverage (+15 percentage points; solid blue line; only for past mass treatment coverage of 50% and 65%). Different panels pertain to increasing pre-control infection levels (top to bottom), and increasing values of past mass treatment coverage (left to right). Grey lines represent smoothed and where relevant extrapolated trendline of simulated outcomes, fitted such that they intersect with the x-axis at the same point as graph lines for annual mass treatment (black solid lines). Values in the corner of each panel represent reductions in remaining program duration (pooled over scenarios for different numbers of past treatment rounds), when increasing coverage (a), switching to 6-monthly mass treatment (b), or switching to 3-monthly mass treatment (c), compared to continuing annual treatment at the same coverage. Panels marked with an asterisk (*) pertain to simulations that did not result in 99% probability of elimination within 20 future treatment rounds, and hence contain no graph lines.
(PDF)

S4 Fig **Predicted minimum remaining program duration required until elimination of onchocerciasis, assuming ivermectin efficacy as in assumption set 1 and high inter-individual variation in exposure to fly bites.** Panels illustrate the minimum remaining program duration (y-axis) required for 99% probability of elimination (absence of infection 50 years after the mass last treatment), given the number of annual mass treatment rounds already completed (x-axis), as predicted by ONCHOSIM (1,000 simulations per scenario). Each panel compares four strategies: continuing annual mass treatment at same coverage (solid black line), switching to 6-monthly mass treatment at same coverage (dashed black line), switching to 3-monthly mass treatment at same coverage (dotted black line), or continuing annual treatment at increased coverage (+15 percentage points; solid blue line; only for past mass treatment coverage of 50% and 65%). Different panels pertain to increasing pre-control infection levels (top to bottom), and increasing values of past mass treatment coverage (left to right). Grey lines represent smoothed and where relevant extrapolated trendline of simulated outcomes, fitted such that they intersect with the x-axis at the same point as graph lines for annual mass treatment (black solid lines). Values in the corner of each panel represent reductions in remaining program duration (pooled over scenarios for different numbers of past treatment rounds), when increasing coverage (a), switching to 6-monthly mass treatment (b), or switching to 3-monthly mass treatment (c), compared to continuing annual treatment at the same coverage. Panels marked with an asterisk (*) pertain to simulations that did not result in 99% probability of elimination within 20 future treatment rounds, and hence contain no graph lines.
(PDF)

S5 Fig **Predicted minimum remaining program duration required until elimination of onchocerciasis, assuming ivermectin efficacy as in assumption set 2 and high inter-individual variation in exposure to fly bites.** Panels illustrate the minimum remaining program duration (y-axis) required for 99% probability of elimination (absence of infection 50 years after the mass last treatment), given the number of annual mass treatment rounds already completed (x-axis), as predicted by ONCHOSIM (1,000 simulations per scenario). Each panel compares four strategies: continuing annual mass treatment at same coverage (solid black line), switching to 6-monthly mass treatment at same coverage (dashed black line), switching to 3-monthly mass treatment at same coverage (dotted black line), or continuing annual treatment at increased coverage (+15 percentage points; solid blue line; only for past mass treatment coverage of 50% and 65%). Different panels pertain to increasing pre-control infection levels (top to bottom), and increasing values of past mass treatment coverage (left to right). Grey lines represent smoothed and where relevant extrapolated trendline of simulated outcomes, fitted such that they intersect with the x-axis at the same point as graph lines for annual mass treatment (black solid lines). Values in the corner of each panel represent reductions in remaining program duration (pooled over scenarios for different numbers of past treatment rounds), when increasing coverage (a), switching to 6-monthly mass treatment (b), or switching to 3-monthly mass treatment (c), compared to continuing annual treatment at the same coverage. Panels marked with an asterisk (*) pertain to simulations that did not result in 99% probability of elimination within 20 future treatment rounds, and hence contain no graph lines.
(PDF)

S1 File **Contains a spreadsheet with the results of all the simulations from the main analysis, along with instructions on how to search and interpret them.**
(ZIP)

S1 Text **Holds a formal description of ONCHOSIM, including details about the model quantification for the sensitivity analysis.** S1 Fig. illustrates the pre-control skin microfilarial density distributions for the different transmission settings used in this study. S2 Fig. illustrates ONCHOSIM predictions for the effect of ivermectin on community infection levels during annual, 6-monthly, and 3-monthly mass treatment, based on two sets of assumptions regarding ivermectin efficacy. S3 Fig. illustrates the minimum remaining program duration required for 99% probability of elimination of onchocerciasis in a setting of low inter-individual variation in exposure to fly bites (as in Fig. 4), but assuming ivermectin efficacy as in assumption set 2 (Table 2). S4 and S5 Figs. are also similar to Fig. 4, though illustrate predictions for settings with high inter-individual variation in exposure to fly bites, assuming ivermectin efficacy according to assumptions set 1 and 2, respectively.
(PDF)
