# Supplementary material for: Elimination of African Onchocerciasis: Modeling the Impact of Increasing the Frequency of Ivermectin Mass Treatment
Source: PLoS One. 2014 Dec 29;9(12):e115886. doi: 10.1371/journal.pone.0115886 (PMC4278850; doi:10.1371/journal.pone.0115886)
Supplement: S2 Fig — ONCHOSIM predictions for community infection levels, based on two sets of assumptions about ivermectin efficacy. Ivermectin was assumed to instantly kill all mf present in an individual. In addition, we assumed either of two alternative sets of assumptions about the effects of ivermectin on adult worms (left and right panels; for details see Table 3). The frequency of ivermectin mass treatment was assumed to be either annual (solid lines), 6-monthly (dashed lines), or 3-monthly (dotted lines). The trends depicted here are the averages of 100 simulations of a hypothetical village with 400 inhabitants and a pre-control community microfilarial load of 30 microfilariae per skin snip. Ivermectin mass treatment was assumed to cover 65% of the population (∼80% of eligible population). (PDF) [file pone.0115886.s002.pdf]

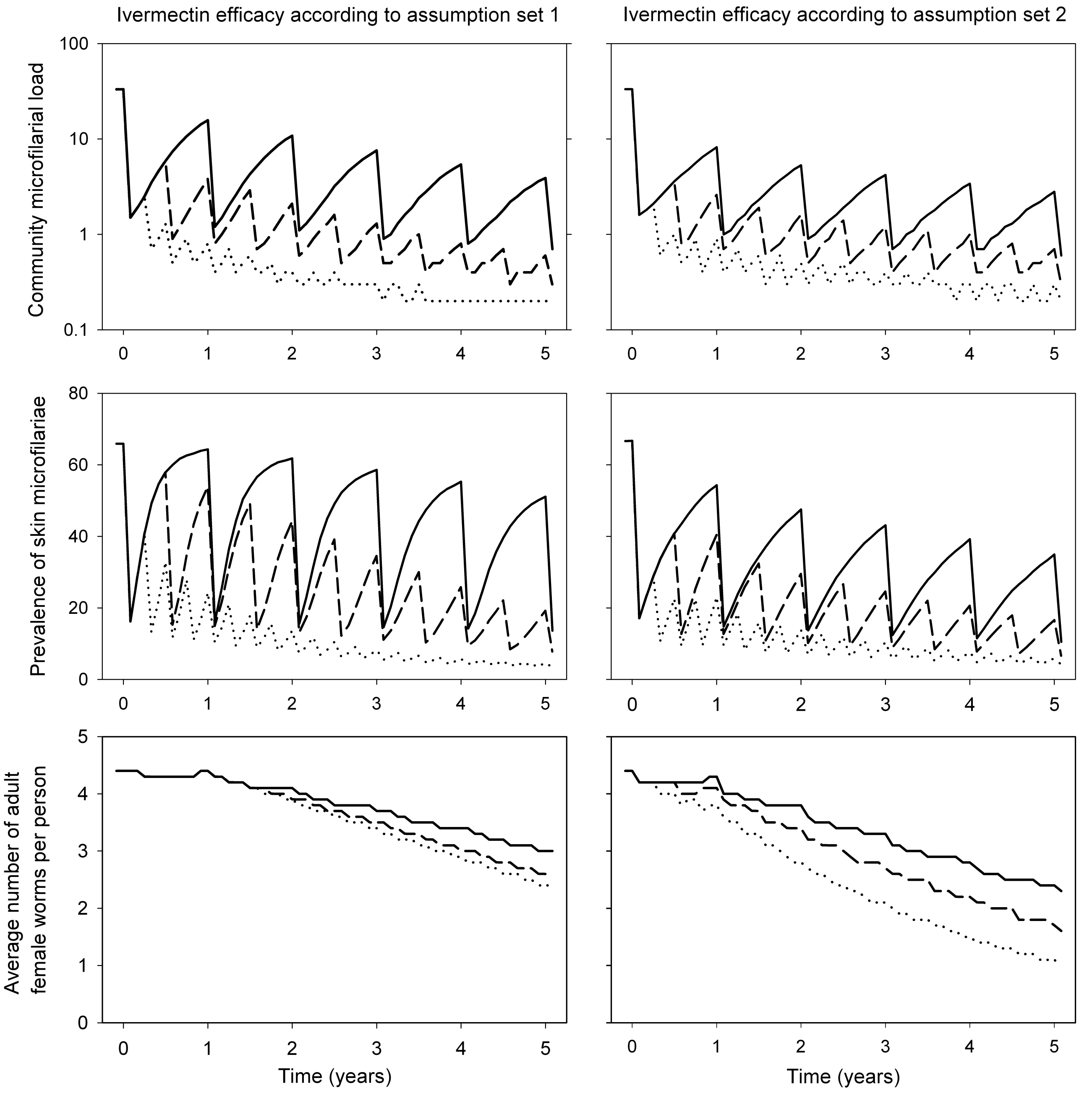

Ivermectin efficacy according to assumption set 1
Ivermectin efficacy according to assumption set 2
Community microfilarial load
100
10
1
0.1
Prevalence of skin microfilariae
80
60
40
20
0
Average number of adult female worms per person
5
4
3
2
1
0
0
1
2
3
4
5
Time (years)
