# Supplementary material for: Elimination of African Onchocerciasis: Modeling the Impact of Increasing the Frequency of Ivermectin Mass Treatment
Source: PLoS One. 2014 Dec 29;9(12):e115886. doi: 10.1371/journal.pone.0115886 (PMC4278850; doi:10.1371/journal.pone.0115886)
Supplement: S5 Fig — Predicted minimum remaining program duration required until elimination of onchocerciasis, assuming ivermectin efficacy as in assumption set 2 and high inter-individual variation in exposure to fly bites. Panels illustrate the minimum remaining program duration (y-axis) required for 99% probability of elimination (absence of infection 50 years after the mass last treatment), given the number of annual mass treatment rounds already completed (x-axis), as predicted by ONCHOSIM (1,000 simulations per scenario). Each panel compares four strategies: continuing annual mass treatment at same coverage (solid black line), switching to 6-monthly mass treatment at same coverage (dashed black line), switching to 3-monthly mass treatment at same coverage (dotted black line), or continuing annual treatment at increased coverage (+15 percentage points; solid blue line; only for past mass treatment coverage of 50% and 65%). Different panels pertain to increasing pre-control infection levels (top to bottom), and increasing values of past mass treatment coverage (left to right). Grey lines represent smoothed and where relevant extrapolated trendline of simulated outcomes, fitted such that they intersect with the x-axis at the same point as graph lines for annual mass treatment (black solid lines). Values in the corner of each panel represent reductions in remaining program duration (pooled over scenarios for different numbers of past treatment rounds), when increasing coverage (a), switching to 6-monthly mass treatment (b), or switching to 3-monthly mass treatment (c), compared to continuing annual treatment at the same coverage. Panels marked with an asterisk (*) pertain to simulations that did not result in 99% probability of elimination within 20 future treatment rounds, and hence contain no graph lines. (PDF) [file pone.0115886.s005.pdf]

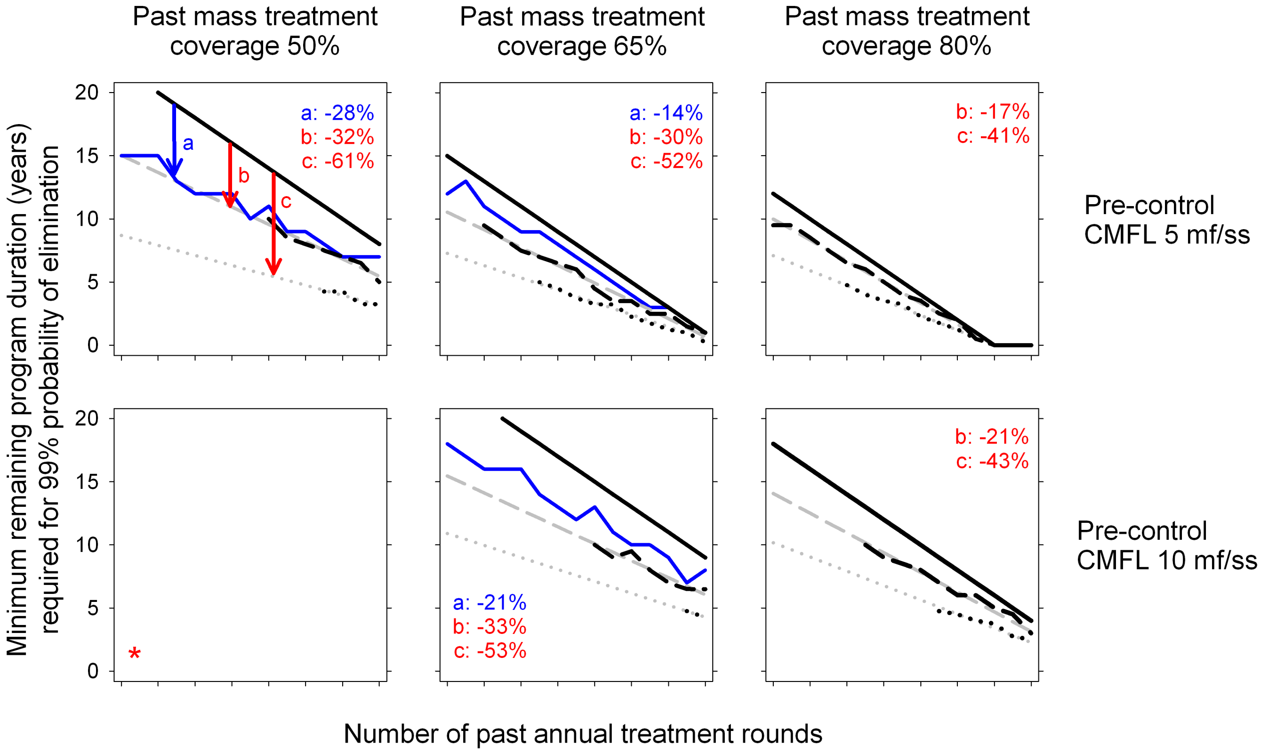

Past mass treatment coverage 50%
Past mass treatment coverage 65%
Past mass treatment coverage 80%
Minimum remaining program duration (years) required for 99% probability of elimination
Number of past annual treatment rounds
Pre-control CMFL 5 mf/ss
Pre-control CMFL 10 mf/ss
a: -28%
b: -32%
c: -61%
a: -14%
b: -30%
c: -52%
b: -17%
c: -41%
a: -21%
b: -33%
c: -53%
b: -21%
c: -43%
a
b
c
*
0
5
10
15
20
