# Supplementary material for: Elimination of African Onchocerciasis: Modeling the Impact of Increasing the Frequency of Ivermectin Mass Treatment
Source: PLoS One. 2014 Dec 29;9(12):e115886. doi: 10.1371/journal.pone.0115886 (PMC4278850; doi:10.1371/journal.pone.0115886)
Supplement: S1 Text — Holds a formal description of ONCHOSIM, including details about the model quantification for the sensitivity analysis. S1 Fig. illustrates the pre-control skin microfilarial density distributions for the different transmission settings used in this study. S2 Fig. illustrates ONCHOSIM predictions for the effect of ivermectin on community infection levels during annual, 6-monthly, and 3-monthly mass treatment, based on two sets of assumptions regarding ivermectin efficacy. S3 Fig. illustrates the minimum remaining program duration required for 99% probability of elimination of onchocerciasis in a setting of low inter-individual variation in exposure to fly bites (as in Fig. 4), but assuming ivermectin efficacy as in assumption set 2 (Table 2). S4 and S5 Figs. are also similar to Fig. 4, though illustrate predictions for settings with high inter-individual variation in exposure to fly bites, assuming ivermectin efficacy according to assumptions set 1 and 2, respectively. (PDF) [file pone.0115886.s007.pdf]

Elimination of African onchocerciasis: modeling the impact of increasing the frequency of ivermectin mass treatment

Luc E. Coffeng[a,*], Wilma A. Stolk[a], Achim Hoerauf[b], Dik Habbema[a], Roel Bakker[a], Adrian D. Hopkins[c], Sake J. de Vlas[a]

[a] Department of Public Health, Erasmus MC University Medical Center Rotterdam, P.O. box 2040, 3000 CA Rotterdam, The Netherlands

[b] Institute of Medical Microbiology, Immunology and Parasitology, University Hospital Bonn, Sigmund Freud Str. 25, 53105 Bonn, Germany

[c] Mectizan Donation Program, 325 Swanton Way, Decatur, Georgia 30030, United States of America

* Correspondence: tel. +31107044269; l.coffeng@erasmusmc.nl, luccoffeng@gmail.com; Erasmus MC University Medical Center Rotterdam, P.O. box 2040, 3000 CA Rotterdam, The Netherlands

Supplementary Text S1: Formal description of ONCHOSIM

Introduction

ONCHOSIM is a computer program for modeling the transmission and control of the tropical parasitic disease onchocerciasis, also known as river blindness. It has been developed in collaboration with the Onchocerciasis Control Programme in West Africa (OCP) and the African Programme for Onchocerciasis Control (APOC) in Central and East Africa, and has been used as a tool in the evaluation and planning of control operations[1–8]. The model simulates the life history of the parasite *Onchocerca volvulus* and of its transmission from person to person by *Simulium* flies. The effects of different control strategies, based on vector control and chemotherapy (e.g. ivermectin), on the transmission and on the disease symptoms can be evaluated and predicted. In the program two simulation techniques are mixed. Stochastic microsimulation is used to calculate the life events of individual persons and inhabitant parasites, while the dynamics of the *Simulium* population and the development of the parasite in the flies are simulated deterministically.

This document

This document gives a complete description of the ONCHOSIM model structure and parameter quantification as used in the simulations for *"Elimination of African onchocerciasis: modeling the impact of increasing the frequency of ivermectin mass treatment"* by Coffeng *et al* (PLoS ONE 2014). Except for the quantification of the effect of ivermectin treatment, the model structure and parameter values presented are the same as reported semi-formally by Plaisier *et al* [1], and formally by Habbema *et al* [4] (the contents of this document are mostly adapted from the latter source). These previous descriptions of the model can be found in the online repository of the Erasmus University Rotterdam.[a] In most simulations we used the default quantification of biological key parameters for savanna type of infection, based on data collected by the Onchocerciasis Control Programme in West

[a] hdl.handle.net/1765/21404

Africa. Where other parameters were used (e.g. for the sensitivity analysis), this is explicitly indicated in the text.

Note that the ONCHOSIM computer program offers the opportunity to change parameter values, to choose other options regarding the type of probability distributions used, and to make structural changes in parts of the model. With footnotes we will highlight alternative options that are not evident from the mathematical description. Table A1 provides an overview of the parameter values used in this study, along with references to sources. For technical details, please refer to the section "Formal description of ONCHOSIM" on page s5.

Software implementation

The current version of ONCHOSIM has been redesigned using object-oriented principles and has been programmed in Java (the original ONCHOSIM was programmed in C++). Individuals and mature worms are modeled as distinct objects. ONCHOSIM is event-driven, which means that time progresses as a result of events (though for most processes, monthly events are used, as in the previous version).

The main advantages of the new implementation are improved code quality and therefore easier maintenance and extension. Several small changes and improvements to model code have already been made, and are named explicitly in the formal description of the model below.

Table A1. ONCHOSIM parameters and their values used in this simulation study.

Parameter	Value	Source
**Demography**		
Human life table (*F(a)*)	See page s5 and Figure A1	[9]
Human fertility (*R(t)*)	See page s5 and Figure A1	[9]
**Exposure**		
Inter-individual variation in exposure to fly bites (*Exi*)	Gamma distribution with mean 1.0 and shape and rate equal to 3.5 or 1.0	[10], unpublished data from OCP
Variation in exposure to fly bites by age and sex (*Exa*)	See page s6	[10]
Seasonal variation in exposure to fly bites (*mbr*)	104%, 91%, 58%, 75%, 75%, 66%, 102%, 133%, 117%, 128%, 146%, and 105% times the average monthly biting rate (January–December)	[11]
**Life history and productivity of the parasite in the human host**		
Worm longevity (*Tl*)	Weibull distribution with mean 10 and shape 3.8 (years)	[2]
Prepatent period	1 year	[2], which refers to [12,13]
Age-dependent potential microfilaria production (*R(a)*)	$R(a) = 0$ for $0 \leq a < 1$<br>$R(a) = 1$ for $1 \leq a < 6$<br>$R(a) = 1-((a-6)/15)$ for $6 \leq a < 21$<br>$R(a) = 0$ for $a > 21$	[2], which refers to [14,15]
Longevity of microfilariae (*Tm*)	9 months	[2]
Worm contribution to the skin mf load (*cw*)	7.6 mf/worm	[10]
Variability in mf per skin snip (2 mg)	Poisson distribution with mean *ss(t)*	[2]
Dispersal factor for worm contribution to skin snip (*d*)	Exponential distribution with mean 1	[2]
Mating cycle (*rc*)	3 months	[2], which refers to [16,17]
Male potential	100 female worms	[2]
**Vision loss**		
Blindness threshold (*Elc*)	Weibull distribution with mean 10,000 and shape 2.0	[7]
Reduction in remaining life expectancy due to blindness	50%	[7], which refers to partly published data from OCP [18]; and [1], which refers to [19,20]
**Parasite and vector**		
Fly survival (*L(t)*)	0.78 flies/day	[4], expert opinion (OCP entomologists)
Gonotrophic cycle (*Pgc(j)*)	$Pgc(j) = 0.0$ for $j \leq 2$ days<br>$Pgc(j) = 0.2$ for $j = 3$ days<br>$Pgc(j) = 0.6$ for $j = 4$ days	[4], expert opinion (OCP entomologists)

Parameter	Value	Source
	Pgc(j) = 0.2 for *j* = 5 days	
	Pgc(j) = 0.0 for *j* ≥ 6 days	
Zoophily (*z*)	4%	[4], expert opinion (OCP entomologists)
Microfilarial uptake (*lu*)	See equation (10); *a* = 1.2, *b* = 0.0213, and *c* = 0.0861 (main analysis); *a* = 1.2, *b* = 0.0213, and *c* = 1.0 (sensitivity analysis)	[8], which refers to [21,22]
Larval development (*F(t)*)	*F(t)* = 0 for *t* ≤ 5 days	[4], expert opinion (OCP entomologists)
	F(t) = 0.07 for *t* = 6 days	
	F(t) = 0.86 for *t* = 7 days	
	F(t) = 1.0 for *t* ≥ 8 days	
Larval survival (*L1-->L3*)	85%	[4], expert opinion (OCP entomologists)
L3 survival (*L3-->L3*)	90%	[4], expert opinion (OCP entomologists)
Larval release (*L3*)	65%	[4], expert opinion (OCP entomologists)
Succes ratio (*sr*)	0.31%	[10,23]
**Mass treatment coverage**		
Coverage (*Cw*)	User-defined	
Age- and sex-specific compliance (*cr(k,s)*)	See page s13	Based on unpublished OCP data
Individual compliance index (co)	Uniform distribution [0,1]	[4]
**Ivermectin**		
Microfilaricidal effect (assumption set 1 and 2)	100%	[3]
Assumption set 1		
Relative effectiveness (*v*)	Weibull distribution with mean 1 and shape 2	[3]
Embryostatic effect (*Tr*, *s*)	11 months	[3]
Reduction in worm fecundity (*d*)	34.9%	[3]
Macrofilaricidal effect	0%	[3]
Assumption set 2		
Embryostatic effect (*tau*)	Exponential distribution with mean 3.5 (years)	This study
Macrofilaricidal effect (male worms)	Beta distribution with mean 0.123 and sample size 50	This study
Macrofilaricidal effect (female worms)	Beta distribution with mean 0.060 and sample size 50	This study
**Larviciding**		
Timing	User-defined	
Coverage	User-defined	

Formal description of ONCHOSIM

Demography

The human population dynamics is governed by birth and death processes. We define *F(a)* as the probability to survive to age *a* (apart from excess mortality due to onchocerciasis related blindness). The values used are as follows:

age (*a*)	0	5	10	15	20	30	50	90
F(a)	1.000	0.804	0.772	0.760	0.740	0.686	0.509	0.000

Survival at intermediate ages is obtained by linear interpolation.

The expected no. of births (per year) at a given moment *t* is given by:

$$R_b(t) = \sum_{k=1}^{n_a} N_f(k,t) \cdot r_b(k) \tag{1}$$

with:

$N_f(k,t)$ no. of women in age group *k* at time *t*

$r_b(k)$ annual birthrate in age-group *k*: 0.109 babies per year for women between 15 and 20 years; 0.300 between 20 and 30 years; 0.119 between 30 and 50 years; 0.0 for all other ages.

n_a no. of age-groups considered.

Each month, $R_b(t)$ is adapted according to the number of women and their age-distribution.[b]

The population distribution resulting from the aforementioned parameters is illustrated in Figure A1, and closely follows the age distribution in Sub-Saharan Africa as estimated by the UN Population Division for the year 2000 (Figure A1) [9].

[b] This is a code improvement compared to the original version of ONCHOSIM, in which the expected number of births was updated annually rather than monthly.

Figure A1. Population demography simulated in ONCHOSIM in absence of excess mortality due to onchocercal blindness (bars), compared to the 2000 population for Sub-Saharan Africa (diamonds; UN Population Division, World Population Prospects: The 2012 Revision).

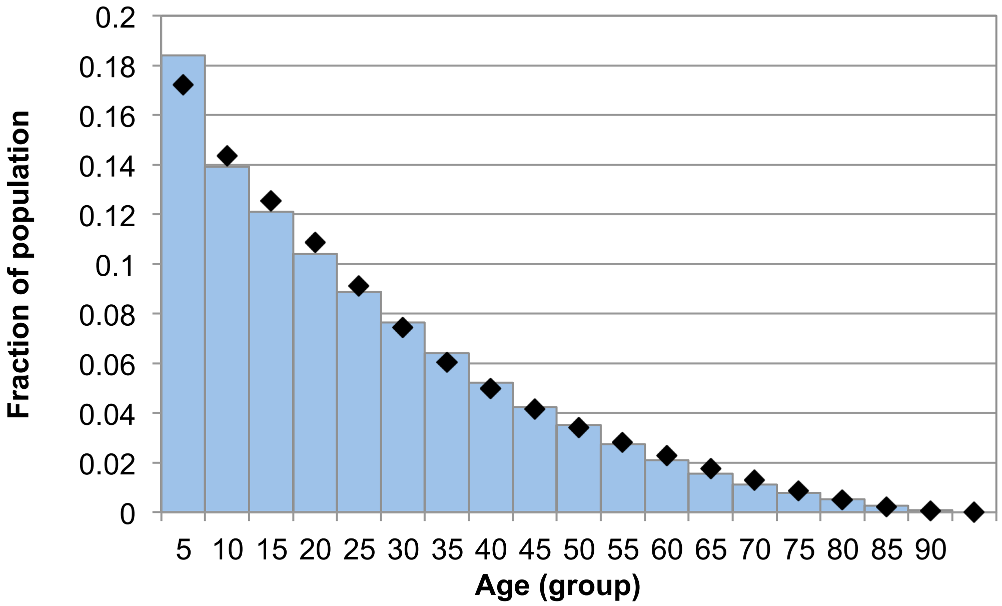

Exposure to blackflies

The number of bites $mbr_i(m)$ a person i gets in month m (in the absence of vector control) is given by:

$$mbr_i(m) = Mbr(m) \cdot Ex_i \qquad (2)$$

with:

Mbr(m) no. bites in month m (m = Jan., Feb., …) for a person with relative exposure 1.

The relative exposure Ex_i is calculated as:

$$Ex_i = Exa(a_i, s_i) \cdot Exi_i \qquad (3)$$

with:

$Exa(a_i,s_i)$ relative exposure of person with age a and sex s: Zero at birth, linear increase between age of 0 and 20 years to 1.0 for men and 0.8 for women, and constant from 20 years onwards.[c]

$Exi_i \sim Gamma(1.0, \alpha_{Exi})$

Exposure index of person i. Exi_i is assumed to follow a gamma distribution with mean 1.0 and shape and rate equal to α_{Exi}. The exposure index of a person remains constant throughout lifetime. For

[c] Alternative functional relationships can be used to describe relative exposure as a function for age. Also empirical functions (specific values for specific age groups) can be given.

selected West African villages (Onchocerciasis Control Programme), estimated α_{Exi} values vary between 1.6 and 12.7.[d]

Mbr(m) values are obtained from six years of fly collections near the village of Asubende (Ghana). There, monthly biting rates of on average 2570 bites per person, varying from 1500 in March to 3750 in November have been found. For the actual biting rates (*Mbr(m)*) inside of this village we multiplied these figures with a factor (called the *relative biting rate*) of 0.95 (note: since we have no measurements of biting rates actually experienced by villagers, we have – arbitrarily – defined a *relative biting rate* of 1.0 – i.e. mean *Mbr* = 2750 – as the biting rate resulting in a geometric mean no. of mf per skin-snip of 100 in hypothetical village with all persons being permanently characterized with a relative exposure of 1.0). Assuming the same seasonal pattern, for other villages relative biting rates have been estimated to vary from 0.4 to 0.9.

Acquisition, development, longevity and productivity of parasites in the human host

If during a blood meal of a fly in month *m* on average *lr* infective larvae are released, the force-of-infection $foi_i(m)$ – defined as the expected number of new adult parasites acquired by person *i* in month *m* – is calculated as:

$$foi_i(m) = mbr_i(m) \cdot lr(m) \cdot sr \tag{4}$$

with:

sr success ratio: fraction of injected L3-larvae succeeding in growing to adult male or female worms: *sr* = 0.0031. An average male:female sex ratio of 1:1 is assumed.

In month *m*, a person *i* is assumed to become infected according to a Poisson process with rate $foi_i(m)$.

The reproductive lifespan of male and female parasites is a random variable: $Tl \sim Weibull(muTl, \alpha_{Tl})$, with mean *muTl* = 10 years and shape α_{Tl} = 3.8.[e] The mf-productivity *r(a,t)* of a female worm of age *a* at time *t* is calculated as follows:

$$r(a,t) = R(a) \cdot m(t) \tag{5}$$

with:

R(a) potential mf-productivity of a female worm of age *a* (in years):

$R(a) = 0$ for $0 \le a < 1$;

$R(a) = 1$ for $1 \le a < 6$;

$R(a) = 1-((a-6)/15)$ for $6 \le a < 21$;

$R(a) = 0$ for $a > 21$.[f]

---

[d] If desired, other continuous probability function can be chosen.

[e] For readers used to the other commonly used parameterization of the Weibull distribution in terms of shape *k* and scale λ, shape *k* is α_{Tl} (as described in this document) and scale $\lambda = muTl / \Gamma(1 + 1 / \alpha_{Tl})$.

[f] Quantifying $R(a) = 0$ for $0 \le a < 1$ is equivalent to assuming an pre-patent period of exactly 1 year for all male and female worms. However, in ONCHOSIM for this pre-patent period other values can be given and it can also

m(t) mating factor at time *t*

To continue mf-production, a female worm must be inseminated each *rc* months (*rc* = reproductive cycle = 3). If insemination took place less than *rc* months ago, then *m(t)* = 1. Otherwise, the probability of insemination or reinsemination $P_{ins}(t)$ in month *t* is given by:

$$\begin{aligned} P_{ins}(t) &= W_m(t)/W_f(t) \quad &\text{if } W_m < W_f \\ P_{ins}(t) &= 1 &\text{if otherwise} \end{aligned} \tag{6}$$

with:

W(t) number of male (W_m) or female (W_f) parasite in the human at time *t*

If no insemination takes place then *m(t)* = 0 and the female worm has a new opportunity in the month *t* + 1. If insemination occurs in month t_i then *m(t)* = 1 during $t_i \leq t < t_i + rc$.[g]

The skin mf-density *sl(t)* at time *t* is calculated by accumulating the mf-production of all female parasites over the past *Tm* months:

$$sl(t) = cw \cdot el(t) \tag{7}$$

$$el(t) = \frac{1}{Tm} \sum_{j=1}^{n_i} \sum_{x=1}^{Tm} r_j(a_j - x, t - x) \tag{8}$$

with:

el(t) the *effective parasite load* at time *t*. This intermediate variable describes the female parasite load obtained by weighting each worm according to the mf-productivity during the past *Tm* months.

cw average contribution of an inseminated worm at peak fecundity (*R* = 1) to the skin mf-density: *cw* = 7.6 mf/worm.[h]

Tm (fixed) microfilarial lifespan: *Tm* = 9 months.

n_i number of parasites alive during at least one of the months *t-1,…,t-Tm*.

Skin-snip count

The expected number of mf in a skin-snip of 2 mg is given by:

$$ss(t) = \frac{cw}{Tm} \sum_{j=1}^{n_i} d_j \sum_{x=1}^{Tm} r_j(a_j - x, t - x) \tag{9}$$

---

be specified as a continuous probability distribution. Also for the potential mf-production after the immature period other values can be chosen.

[g] In ONCHOSIM we have one additional parameter to influence the mating probability P_{ins}. This parameter is called *male potential* and is multiplied with the male:female worm sex ratio. Assigning a high value to this *male potential* (e.g. 100) implies that mating (if required) will always take place if there is at least one adult male worm. In case of a negative *male potential* female worms can produce Mf in the absence of male worms.

[h] Instead of a linear relationship between *sl* and *el* other functional relationships can be chosen (e.g. a saturating function).

with:

d_j *dispersal factor* of female parasite *j*. This is a random variable drawn for every "newborn" worm, and accounts for differences in the contribution of female worms to the mf-density at the standard site of the body where snips are taken (hip). We assume that d_j follows an exponential: $d_j \sim Expo(1.0)$.

The actual number of mf per skin-snip follow a Poisson distribution: $ss_{obs}(t) \sim Poisson(ss(t))$.[i] At each epidemiological survey 2 snips are taken from all simulated persons.[j] The results of such a survey are post-processed to arrive at age and sex-specific and standardized mf prevalences.

Uptake, development and release of larvae in the vector

On the basis of fly-feeding experiments in OCP the following expression for the relation between L1-uptake (*lu*) and skin-microfilarial density (*sl*) has been derived (note: since most of the mf engorged during a blood meal are trapped in the fly, we consider 'L1-uptake' rather than mf-uptake):

$$lu = a \cdot \left(1 - e^{-b \cdot sl}\right) \cdot \left(1 + e^{-c \cdot sl}\right) \tag{10}$$

with:

$a = 1.2$, $b = 0.0213$, and $c = 0.0861$ (the initial slope of this relationship equals $2ab$).[k]

The mean L1-uptake in the fly population per fly bite in month *m* is now calculated as:

$$\overline{lu}(m) = \sum_{i=1}^{N(m)} \left(Ex_i \cdot lu_i\right) / \sum_{i=1}^{N(m)} Ex_i \tag{11}$$

with:

$N(m)$ No. of persons in month *m*.

It is assumed that a fixed proportion of the L1-larvae develops to the L3—stage and will be released at one of the subsequent bites:

$$lr(m) = v \cdot \overline{lu}(m) \tag{12}$$

with:

---

[i] Or any other discrete probability function (e.g. geometric).

[j] Or any other number.

[k] Other functional relationships can also be defined. In the sensitivity analysis, we set $c = 1.0$ to simulate a situation with less pronounced negative density dependence in transmission (which may be reflective of the situation in forest areas). This alternative parameter value results in a less concave shape of the function, while the slope in the origin (which equals $2ab$) and the final saturation level (a) remain the same. In terms of L1-uptake, this means that uptake is up to 40% lower for skin mf densities <10 mf/ss, and nearly unchanged for skin mf densities >40 mf/ss. Increases in c beyond 1.0 do not affect the shape of the function very much. The choice of setting the value $c = 1.0$ was arbitrary, and does not necessarily represent forest vector-parasite complexes. This hypothetical case is included to show the importance of density dependence assumptions.

lr(m) mean L3-release per bite in month *m*

v transmission probability: average probability that an L1-larva is release as an infective larva.

The calculation of the transmission probability *v* is complicated. In calculating *v* we take into account the life history of the fly starting from her first blood meal. We assume that blood meals are taken at fixed hours during daytime, so that we can use 1 day time steps. Though we take into account differences in the length of gonotrophic cycle between flies, in the model we assume that a particular fly has always the same cycle length (which equals the time between two successive blood meals). We further explicitly account for variation in the duration of development from L1 to L3. the basic assumption underlying the use of a fixed proportion *v* is that at any moment the fly-population has a stable age-distribution and that the no. of bites per person is large enough to disregard the age of the biting flies.

Calculation of the transmission probability *v*

For ONCHOSIM-2, transmission probability *v* has to be calculated outside the model and has to be given as a parameter. This section describes the necessary calculations.

Assume that a fly engorges one L1-larva at her m^{th} blood meal, then the probability to release an L3-larva *n* blood meals later is by:

$$P_{rel}\left(n \mid i,j,m\right) = P_{L1\to L3} \cdot \left(1 - P_{L3\to}\right)^{i} \cdot P_{L3\to L3}^{i} \cdot P_{L3\to} \cdot S\left(m, n \cdot j\right) \tag{13}$$

with:

$P_{rel}(n|i,j,m)$

The probability to release one L3 larva at the $(m+n)^{th}$ blood meal if one L1 larva has been engorged at the mth blood meal, given that

- a gonotrophic cycle takes *j* days
- between blood meals *m* and *m* + *n* there have been *i* potentially infective blood meals (i.e. blood meals at which the L1-larva had already developed to the L3-stage)

$P_{L1\to L3}$ The probability that an L1-larva develops to the L3-stage, given survival of the fly: $P_{L1\to L3} = 0.85$.

$P_{L3\to L3}$ The probability that an L3-larva which is not released at a given blood meal survives to a next blood meal, given survival of the fly: $P_{L3\to L3} = 0.90$.

$P_{L3\to}$ The probability that an L3-larva is released at a blood meal: $P_{L3\to} = 0.65$.

S(m,t) The probability that a fly survives for *t* days until blood meal *m*.

In order to arrive at a general solution for all possible values of *i*, we use the probability distribution of the number of potentially infective blood meals since the intake-meal and before the release meal:

$$P_{rel}\left(n \mid j,m\right) = \sum_{i=0}^{n-1}\left[P_{rel}\left(n \mid i,j,m\right) \cdot P_{ib}\left(i \mid n,j\right)\right] \tag{14}$$

$$P_{ib}\left(i \mid n,j\right) = F_{dL1\rightarrow L3}\left(j\left(n-i\right)\right) - F_{dL1\rightarrow L3}\left(j\left(n-i-1\right)\right) \quad (15)$$

with:

$P_{ib}(i \mid n,j)$

The probability that before the nth blood meal since intake, i blood meals have been potentially infective (L1 has become L3), given a cycle length of j days.

$F_{dL1\rightarrow L3}(t)$

Probability that the duration of development of L1 to L3 is equal to or less than t days ($F_{dL1\rightarrow L3}(t)$ = 0.0 for $t \leq 5$; 0.07 for $t = 6$; 0.86 for $t = 7$; 1.0 for $t \geq 8$ days).

A general solution for all possible values of m can be obtained by incorporating the probability that a fly takes her m^{th} blood meal:

$$P_{rel}\left(n \mid j\right) = \sum_{m=1}^{m_{\max}} \left[P_{rel}\left(n \mid j,m\right) \cdot P_b\left(m \mid j\right)\right] \quad (16)$$

$$P_b\left(m \mid j\right) = L\left(j\left(m-1\right)\right) / \sum_{m=1}^{m_{\max}} \left(j\left(m-1\right)\right) \quad (17)$$

with:

$P_b(m \mid j)$

Probability that a feeding fly takes her m^{th} blood meal at a cycle length of j days.

$L(t)$ Probability that a fly lives for at least t days. At present we assume an age-independent daily survival of 0.78.

Generalizing for j can be achieved by summation, weighted for the probability distribution of the duration of the gonotrophic cycle:

$$P\left(n\right) = \sum_{j=j_{\min}}^{j_{\max}} \left[P_{rel}\left(n \mid j\right) \cdot P_{gc}\left(j\right)\right] \quad (18)$$

with:

$P_{gc}(j)$ Probability that a gonotrophic cycle takes j days (i.e. j days between successive blood meals; $P_{gc}(j)$ = 0.0 for $j \leq 2$; 0.2 for $j = 3$; 0.6 for $j = 4$; 0.2 for $j = 5$; 0.0 for $j \geq 6$ days).

Using the following equality

$$S\left(m,n \cdot j\right) = L\left(j\left(m+n-1\right)\right) / L\left(j\left(m-1\right)\right) \quad (19)$$

the average probability than an L1-larva taken from a human will develop to the L3-stage and released to another human is given by:

$$P_{rel} = P_{L1\rightarrow L3} \cdot P_{L3\rightarrow} \cdot$$
$$\sum_{j=j_{\min}}^{j_{\max}} \left\{ \begin{array}{l} P_{gc}(j) \cdot \sum_{m=1}^{m_{\max}} \left[\frac{1}{\sum_{m=1}^{m_{\max}} L(j(m-1))} \right] \cdot \sum_{n=1}^{n_{\max}} \left\{ L(j(m+n-1)) \cdot \sum_{i=0}^{n-1} \left[(1-P_{L3\rightarrow}) \cdot P_{L3\rightarrow L3} \right]^i \cdot \right. \\ \left. \sum_{i=0}^{n-1} \left[(1-P_{L3\rightarrow}) \cdot P_{L3\rightarrow L3} \right]^i \cdot \left[F_{dL1\rightarrow L3}(j(n-i)) - F_{dL1\rightarrow L3}(j(n-i-1)) \right] \right\} \end{array} \right\} \quad (20)$$

In equation (16), (17) and (18):

$$m_{\max} = \frac{a_{\max}}{j} + 1 \quad \text{, truncated to integer}$$
$$n_{\max} = \frac{a_{\max} - (m \cdot j)}{j} + 1 \quad \text{, truncated to integer} \quad (21)$$

with:

a_{max} Maximum attainable age of the fly (i.e. age at which *L(T)* approaches zero).

The transmission probability *v* is now given by:

$$v = P_{rel} \cdot (1 - z) \quad (22)$$

with:

z Fraction of fly-bites on non-human objects (zoophily; *z* = 0.04)

Using the indicated quantifications, we have calculated a *v* of 0.073 released larvae per L1-larva resulting from a given mf-uptake. Note that formula (20) reduces to a much more simple form if we assume that each day a fraction *S* of the flies survive, that the gonotrophic cycle has a fixed duration of *dgc* days, and that the number of blood meals needed to complete the development of L1 to L3 is fixed to $n1\rightarrow 3$:

$$P_{rel} = P_{L1\rightarrow L3} \cdot P_{L3\rightarrow} \cdot \frac{S^{n1\rightarrow 3 \cdot dgc}}{1 - S^{dgc} \cdot (1 - P_{L3\rightarrow}) \cdot P_{L3\rightarrow L3}} \quad (23)$$

Blindness and excess mortality

The event of a person going blind at age *a* (months) depends on the *accumulated parasite load (elc)* of a person:

$$elc(a) = \sum_{x=o}^{a} el(x) \quad (24)$$

Each person has a threshold level *elc* (denoted as *Elc*) at which a person goes blind. *Elc* follows a probability distribution: $Elc \sim Weibull(muElc, \alpha_{Elc})$, with mean *muElc* = 10,000 and shape α_{Elc} = 2.0. Person *i* goes blind at age *a* when:

$$elc_i(a) \geq Elc_i > elc_i(a-1) \quad (25)$$

At that moment the remaining lifespan at age *a* is reduced by a factor *rl* which follows a uniform distribution on [0,1] (hence on average *rl* = 0.5).[l]

Ivermectin: mass treatment coverage and compliance

The primary characteristic of a certain ivermectin mass treatment *w* is the coverage C_w (fraction of the population treated; typically 0.65). However, a difficulty in calculating individual chances of participation is that there are several exclusion criteria for the drug. Moreover, compliance to treatment differs from person to person. Exclusion criteria can be either permanent (chronic illness) or transient (children below 5 and pregnant or breast-feeding women). We define the eligible population as the total population *minus* a fraction *fc* (=0.05) that is permanently excluded from treatment (in the model from birth to death). The coverage among the eligible population is now given by:[m]

$$C'_w = C_w / \left(1 - fc\right) \tag{26}$$

The transient contra-indications and other age- and sex-related factors are taken into account in the age- and sex-specific relative compliance $c_r(k,s)$ for each age-group *k* and sex *s*. Based on OCP data we use:

age-group (*k*)	0-4	5-9	10-14	15-19	20-29	30-49	50+
$c_r(k,males)$	0.00	0.75	0.80	0.80	0.70	0.75	0.80
$c_r(k,females)$	0.00	0.5	0.70	0.74	0.65	0.70	0.75

Note that in $c_r(k,s,)$ only the *ratio* between the values for the different groups is relevant.

Now, the coverage $c(k,s,w)$ in each of the age- and sex-groups at treatment round *w* is calculated as:

$$c\left(k,s,w\right) = \frac{c_r\left(k,s\right) \cdot N\left(w\right)}{\sum_{s=1}^{2} \sum_{k=1}^{n_a} c_r\left(k,s\right) \cdot N\left(k,s,w\right)} \cdot C'_w \tag{27}$$

with:

N(k,s,w,)

Number of individuals eligible to treatment in age-group *k* and sex *s* at treatment round *w*.

N(w) Total number of eligible individuals at treatment round *w*.

Finally, the probability to participate in treatment round *w* for an eligible person *i* of age-group *k* and sex *s* is given by:

[l] Any other probability distribution defined on [0,1] can be used (e.g. a beta distribution).

[m] This is a code improvement compared to the original version of ONCHOSIM, in which the expected coverage in the eligible population was defined as $C'_w = C_w + fc$. This code improvement did not have consequences for the quantification of the effects of ivermectin, as these were calibrated to data from individuals who had been treated with certainty.

$$Ptr_{i,w} = co_i^{\frac{1-c(k,s,w)}{c(k,s,w)}} \tag{28}$$

with:

co_i Personal compliance index. This is considered as a lifelong property and is generated by a uniform distribution on [0,1]

Note that for all k and s the average value of $Ptr_{i,w}$ equals $c(k,s,w)$.[n]

Ivermectin: the parasitological effect of treatment

In the present simulation study, we use two alternative sets of assumptions about ivermectin efficacy. The first assumption set was calibrated on community trial data [3,11] and has been used in previous applications of the model [5–7]. The second assumption set was developed for this particular study, and tries to capture more recent insights into the mechanisms by which ivermectin affects adult worms through macrofilaricidal effects and congestion of female worm uteri with dead mf [24–34]. Assumption set 1 was adopted directly from the original publication on its quantification[3]. Assumption set 2 was calibrated using aggregate data from Guatemala [27] and literature data from a published meta-analysis [35] (explained in more detail at the end of this document). Table 2 in the main document gives an overview of the quantification of the two assumption sets. Here, we give a mathematical description of the model mechanics. A detailed description of the methods used to calibrate assumption set 2 is given at the end of this documents (methods for quantifying assumption set 1 have been described elsewhere [3]).

Assumption set 1

In this set of assumptions, we assume that an effective treatment with ivermectin causes elimination of 100% of the microfilariae from the skin-tissues.[o] In addition, the first assumption set assumes that ivermectin permanently and cumulatively decreases the capacity of adult female worms to produce mf, after a temporal interruption in mf productivity. The temporal and permanent impact of the drug on the subsequent mf productivity r of a female parasite j in person i is given by:

$$\begin{aligned}
r_{j,i}(t) &= r^0_{j,i}(t)\cdot(1-v_i d)\cdot\left(\frac{t}{v_i Tr}\right)^s && \text{if } u_j > v_i m,\quad v_i d < 1,\quad \text{and } t < v_i Tr \\
r_{j,i}(t) &= r^0_{j,i}(t)\cdot(1-v_i d) && \text{if } u_j > v_i m,\quad v_i d < 1,\quad \text{and } t \geq v_i Tr \qquad (29) \\
r_{j,i}(t) &= 0 && \text{otherwise}
\end{aligned}$$

with:

t Time (months) since treatment.

---

[n] In ONCHOSIM we recognize 3 ‘coverage-models’. In model 0, the probability to be treated is as given in formula (28). In model 1, the probability is equal to $c(k,s,w)$ (hence, the ‘compliance index’ is ignored). The simplest model is model 2 in which the treatment probability simply equals C'_w. All models take account of a fraction fc of permanently excluded persons.

[o] Lower fractions are also possible. Further, the instantaneous effect can be described by a continuous probability distribution (which should generate random variables between 0 and 1).

$r_{j,i}(t)$ mf-productivity of female worm *j* at *t* months after treatment with ivermectin of person *i* (see equation (5)).

$r^0_{j,i}(t)$ The mf-productivity of this worm *j* had person *i* not been treated at the last round.

v_i Relative effectiveness of treatment in person *i*. For every separate treatment and person, a new value is drawn for v_i (i.e. the relative effectiveness applies to all worms in a person during a specific treatment).

d Average permanent (unrecoverable) reduction in mf-productivity resulting from treatment (d = 0.349).[p]

Tr Average duration of the period of recovery, i.e. the period during which the mf-productivity of the female worm increases from 0 to the new equilibrium (*Tr* = 11 months).

s Shape parameter of the recovery function (*s* = 1.5).

u_j Random number on [0,1] generate for each female worm *j*.

m Average fraction of female worms killed as a result of treatment (in ivermectin assumption set 1, *m* = 0).

The relative effectiveness v_i is a random variable generated by a probability distribution: $v_i \sim Weibull(1.0, \alpha_v)$, with $\alpha_v = 2.0$. In addition to this, we explicitly consider that some persons (5% of the treated population) do not at all react to the drug during a certain treatment due to malabsorption (e.g. due to vomiting or diarrhoea).[q]

Assumption set 2

In this assumption set 2, we also assume that an effective treatment with ivermectin causes elimination of 100% of the microfilariae from the skin-tissues. We assume that ivermectin does not permanently reduce mf production capacity, but rather kills a proportion of all female and male worms in a person, allowing the expected proportion to differ by worm gender. The proportions of female and male worms killed (p_f and p_m) are described by beta distributions, allowing variation between persons and treatments (but not between worms within persons):

$$\begin{aligned} p_f &\sim Beta\left(a_f, b_f\right) \\ p_m &\sim Beta\left(a_m, b_m\right) \end{aligned} \tag{30}$$

where:

$$\begin{aligned} a_x &= \mu_x \cdot W \\ b_x &= \left(1-\mu_x\right) \cdot W \end{aligned} \tag{31}$$

with:

a_x, b_x Shape parameters for a beta distribution with mean $a_x / (a_x + b_x)$ (which is equal to μ_x) and variance $(a_x b_x) / ((a_x + b_x)^2 (a_x + b_x + 1))$.

[p] In the sensitivity analysis, this parameter was set to a 2/3 and 3/2 higher value.

[q] Apart from the permanent reduction in mf-production (*d*) of female worms, another irreversible effect on (male + female) worms that can be specified is a fraction of the worms that is killed immediately after treatment.

μ_x Expected proportion of worms of gender x killed. For females worms, this parameter was calibrated at 0.060 (6.0%); for male worms, this was value was 0.123 (12.3%).[r]

W Sample size of the beta distribution. The larger W is, the smaller the variation in macrofilaricidal effect between treatments and persons. Because W could not be estimated from the data, it was arbitrarily set to 50, such that the 2.5% and 97.5% percentiles of the proportion of female and male worms killed were 3.9% – 19.0% and 1.3% – 14.0%, respectively.

We assumed that the macrofilaricidal effects on male and female worms were perfectly correlated by drawing a random number from the [0,1] interval and feeding these into the inverse cumulative beta distributions for p_f and p_m.

In addition, assumption set 2 specifies that successful treatment causes a temporary stop in mf production by female worms due to uteral congestion with dead mf. This effect only takes place if a female worm was actually producing mf at the time treatment. Time until resumption of mf production is assumed to vary per worm and treatment, following an exponential distribution with a mean of 3.5 years. This implies that 5% of adult female worms can be inseminated and release microfilariae within two months after exposure to ivermectin, 63% after 3.5 years, and 95% after 10.5 years. In general, the fraction of adult female worms with resolved congestion is given by:

$$f_r(t) = 1 - e^{-t/\tau} \tag{32}$$

with:

f_r The fraction of adult female worms in which uteral congestion has resolved.

τ The mean of the exponential distribution.

Vector control

Vector control is modeled as a % reduction of the monthly biting rates during a given period of time. A period of vector control[s] is specified as the year + month of the beginning of the strategy and the year + month of the end of a strategy. If a certain month during a period of d days larvicides have been applied, then the reduction in *Mbr(m)* in that month equals d/30 x 100%.

Simulation warm-up

In general, before starting simulation of interventions in ONCHOSIM, a 200-year warm-up period is simulated, such as to allow the human and worm population to establish equilibrium levels, given the parameters for average fly biting rate and inter-individual variation in exposure to infection. At the start of the warm-up period, an artificial force of infection of four L3 larvae per person per year is simulated for 90 months (7.5 years), allowing worms to establish themselves in the human population. After those initial 90 months, transmission is governed exclusively by fly bites and the processes described above. After the 200 warm-up

[r] In the sensitivity analysis, these proportions were set to 2/3 lower and 3/2 higher values.

[s] In ONCHOSIM more than on period of vector control, each with its own effectiveness can be specified.

years, the simulated infection levels are no longer correlated with the initial conditions at the start of the warm-up period.

Calibration of assumption set 2 regarding ivermectin efficacy

The model parameters for assumption set 2 were calibrated in several steps. Parameters could not be estimated all at once because there was high correlation between the parameters for excess mortality among worms and duration until resolution of the plug of dead microfilariae. This correlation can be explained as follows: long persistence of the plug is essentially equivalent to mortality, because either way, worms can no longer produce microfilariae. Therefore, we first estimated mortality among worms separately from Guatemalan data on worm survival, published by Cupp [27] . Next we estimated the mean and variance of the duration until resolution of the plug of dead microfilariae, based on a published meta-analysis on the effects of a single dose of ivermectin [35].

Calibration of macrofilaricidal effects

The parameter values for macrofilaricidal effects of ivermectin were estimates in two steps. First, we roughly estimated excess mortality among male and female worms by means of a statistical model. Next, we refined our estimates by dynamically modeling worm survival in ONCHOSIM. Estimates were based on data published Duke *et al* [31], and republished by Cupp *et al* [27]. The data pertain to a field study on worm survival as observed in extirpated nodules from infected volunteers. The volunteers all lived in a community where semiannual treatment was taking place, and a selection of volunteers received three monthly treatments. Worm counts were compared between two treatment arms: quarterly treated individuals and untreated controls from the area. Data were available for one, two, and three years after start of mass treatment. Nodules were extirpated at the same time points for both treatment arms (Table A2).

When analyzing these data, we assumed that during the trial, minimal transmission of L3 took place, which is likely as it was reported that most people in the community took ivermectin. In other words, we attribute the observed trends in number of worms entirely to natural attrition and excess mortality from ivermectin. We only considered the ratio of the average number of (male or female) live worms in the treatment arm over the control arm, at each time point, assuming that natural attrition is the same for the two treatment arms. We estimated excess mortality among male and female worms by maximizing the log likelihood function, assuming a Bernoulli model (with proportions as observations instead of zeroes and ones) and equal weight for all data points:

$$\sum_{i=1}^{3} p_i \cdot \log(\hat{p}_i) + (1-p_i) \cdot \log(1-\hat{p}_i) \quad (33)$$

Here, *p-hat* is the estimated ratio of number of worms in the quarterly and control treatment arms (assuming that this ratio is always a proportion, i.e. there will always be fewer worms in the quarterly treatment arm), *p* is the observed ratio, and *i* is the *i*-th data point (three time points for each sex). The estimated ratio of number of worms in the two treatment arms *p-hat* was calculated by

$$\hat{p}_i = (\hat{m}_{sex})^{Tq_i} / (\hat{m}_{sex})^{Tc_i} = (\hat{m}_{sex})^{Tq_i - Tc_i} = (\hat{m}_{sex})^{Tq_i} \quad (34)$$

Here, *m-hat* is one minus the estimated excess mortality rate for male or female worms, and T_{qi} and T_{ci} are the cumulative number of treatments at time point *i*. Natural attrition is not part of this equation, as we assume that it is equal for both treatment.

Based on all three time points, excess mortality among male worms was estimated to be 14.0%, for female worms this was estimated at 6.8%. However, it is possible that during that first year after the first treatment, in reality there was still an influx of adult worms from the pre-patent worm population that was transmitted in the previous year. Assuming that the pre-patent period is about one year (as in ONCHOSIM), we also estimated excess mortality based on the second and third time points only: 15.0% and 7.3% among male and female worms, respectively (Table A2).

Table A2: Estimates of excess mortality among male and female worms based on worm survival data from Guatemala [27,31]. We assumed that no transmission of L3 took place during periods of 6-monthly and 3-monthly mass treatment. All data points were treated with equal weight.

**Time (years)**	**1 yr**			**2 yr**			**3 yr**		
**Treatment arm***	**Control**	**4x/year**	**Ratio**	**Control**	**4x/year**	**Ratio**	**Control**	**4x/year**	**Ratio**
Number of treatments	0	4		0	8		0	11	
Female worms per nodule	1.54	1.26	0.818	1.58	1.08	0.684	2.00	0.61	0.305
Male worms per nodule	1.29	0.80	0.620	1.17	0.25	0.214	1.30	0.28	0.215
**Estimated excess mortality (fraction) per treatment****	**Statistical model**			**Dynamical model (ONCHOSIM)**					
Females worms	0.073	(0.068)		0.060	(0.060)				
Male worms	0.150	(0.140)		0.123	(0.123)				
total -2LL	4.742			2.601					

* Control subjects (C) were untreated, but came from the same population as the quarterly (4x/year) treated individuals. Both treatment arms originated from a population undergoing semiannual treatment. Therefore, we assume that no (minimal if any) transmission of infection took place during the trial. ** Values between brackets pertain to an analysis of data from all three time points. However, at the first time point, there was probably still influx of new adult worms from the pre-patent population that was transmitted in a previous year. Therefore, the first time point was excluded from analysis.

To refine above statistical estimates, we also predicted number of adult female worms per person for each treatment arm in ONCHOSIM (ONCHOSIM currently does not provide output on male worms). The number of adult female worms per person was assumed to be proportional to the number of female worms in an extirpated nodule from such persons (nodule extirpation is currently not implemented in ONCHOSIM). Ratios of number of female worms in the simulated treatment arms were then compared to the original data. For these simulations, we modeled a human population exposed to three L3 stage larvae per person per year, allowing for variation by age, sex, and occupation/behaviour (equivalent to CMFL ~55 mf/ss). The infection level was set this high to allow a large number of worms to be simulated. The actual infection level was assumed to be of no consequence for excess mortality among worms. The trial was modeled such that after the first treatment, there was no more transmission in either treatment arm (i.e. force of infection equal to zero). Again, predicted ratios of live female worms in the treatment arms were compared to the data pertaining to either all three time points, or to the second and third time point only. In both comparisons, the best model fit was obtained with 6% excess mortality among female worms. Because ONCHOSIM currently does not provide output on the number of male worms, we assumed that excess mortality among male worms is proportional to that among female worms (i.e. 6 * 15 / 7.3 = 12.3%).

The fit of the ONCHOSIM predictions to the data did not change in an informative way too allow calibration of the parameter for variation in the macrofilaricidal effect per treatment and individual. Therefore, the sample size of the beta distribution was arbitrarily set to 50.

Calibration of parameters for duration of uteral congestion in female worms

In general, parameter calibration for the duration of uteral congestion by dead mf was performed by means of a grid search of parameter values. Exploratory simulations for combinations of parameter values from a rough grid were used to identify the most likely ranges for parameter values. More detailed grid searches spanning a smaller range of parameter values were used to fine-tune parameter values. Model fit was optimized by maximizing the log likelihood for observed relative infection levels (observed levels / pre-control levels) versus predicted relative infection levels. Excess mortality among female and male worms was assumed to be 6.0% and 12.3%, respectively, based on the analysis described in the previous section.

Parameters for the mean and variation of plug longevity were calibrated to data from a meta-analysis of the effect of a single dose of ivermectin by Basanez *et al* [35]. We discarded data pertaining to the period up to one month after treatment with ivermectin. These data show a declining pattern in mf loads in the skin, whereas in ONCHOSIM, we assume that microfilariae are instantly killed by ivermectin. This discrepancy is probably not relevant for any transmission effects in the long term, but would affect model fit if the data were included. Because almost all studies included in the meta-analysis reported data pertaining to geometric microfilarial loads in individuals of at least age 20, we compared data to model predictions for CMFL (geometric mean load in people of age 20 and above). To reduce the influence of sources of variation between studies, comparisons were made in terms of CMFL relative to the pre-control level of CMFL (similar to the methodology in the meta-analysis). Almost all study populations had geometric mean microfilarial loads between 30 and 60 mf/ss. From exploratory simulations we concluded that for endemic populations with a CMFL of ~30 to ~60, the pre-control CMFL hardly influences trends in relative CMFL (relative to pre-control CMFL) during two years after a single dose of ivermectin. Therefore, we assumed pre-control CMFL to be 50 mf/ss for all data points (equivalent to a stable force of infection of about three stage L3 larvae per person per year). Exploratory simulations showed that the magnitude of variation in exposure to force of infection at the level of individuals (i.e., variation related to behaviour/occupation) influenced the simulated pre-control CMFL, but not simulated trends in relative CMFL over time (relative to pre-control CMFL). Therefore, we assumed that variation at the level of individuals was the same for all data points. Relative exposure at the individual level was assumed to follow a Gamma distribution with mean 1 and shape 3.5; the value 3.5 having been previously estimated to be appropriate for describing a mixed population consisting of individuals from several villages (unpublished data).

We assumed that there were no effects of ivermectin on transmission (force of infection) because most studies pertained to clinical trials (e.g. no population-level effects of ivermectin), and because transmission effects of a single dose of ivermectin – if any – only come into effect after at least one year (the estimated pre-patent period for a new-born worm). All data points pertained to the first year after treatment with ivermectin, except for one clinical trial that reported results for 22 months post-treatment. To nullify transmission effects in the simulations, we set the fly biting rate to zero and artificially introduced an average of three L3 larvae per person per year into the simulated population (allowing for variation by age, sex, and individual behaviour/occupation). To mimic a trial setting in the ONCHOSIM simulations, we assumed that all adult individuals took ivermectin (100% compliance). The only reason that treatment was assumed to be able to fail, was through malabsorption of

ivermectin (in 5% of individuals, an assumptions already implemented in ONCHOSIM). Further, sensitivity analyses showed that proportion of male worms killed by ivermectin and variation in killing by treatment and person were not important for fitting the model to the data (these effects may only become important after repeated treatments).

The model parameters were fit by maximizing the following likelihood, assuming a Bernoulli model (with proportions as observations instead of zeroes and ones) and equal weights for all data points:

$$\sum_{i=1}^{n} p_i \cdot \log(\hat{p}_i) + (1 - p_i) \cdot \log(1 - \hat{p}_i) \qquad (35)$$

Here, *p-hat*$_i$ is the estimated relative CMFL level (ONCHOSIM), p_i is the observed relative CMFL level (meta-analysis data), and n is the number of data points. We assumed equal weights for all data points.

We first performed exploratory simulations for a low resolution grid of parameter values to determine the most likely range for each parameter; the parameter for mean duration was varied by 12 months and the parameter for the shape of the Weibull distribution of duration was given the values 0.5, 1, 2, 4, 8, and 16. From these simulations, we concluded that the shape parameter for the Weibull distribution for plug longevity should be about 1. Larger values forced the pattern in CMFL over time after treatment to follow a sigmoidal pattern, yielding worse fit of the model to the data. Smaller values caused CMFL to increase quickly and steeply after treatment and reach a plateau state. In both cases, the model predicted CMFL levels that were generally higher than those observed in the data. Therefore, we simply assumed that the shape parameter was 1, making the Weibull distribution equivalent to an exponential distribution, and yielding a quasi-linearly increasing pattern of CMFL over time after treatment (Figure A2).

Figure A2: ONCHOSIM predictions for prevalence of microfilariae in the skin (mf prevalence) and the community microfilarial load (CMFL; geometric mean skin microfilarial load in individuals of age 20 and above) during two years after a single dose of ivermectin, relative to pre-control levels of infection. Data points

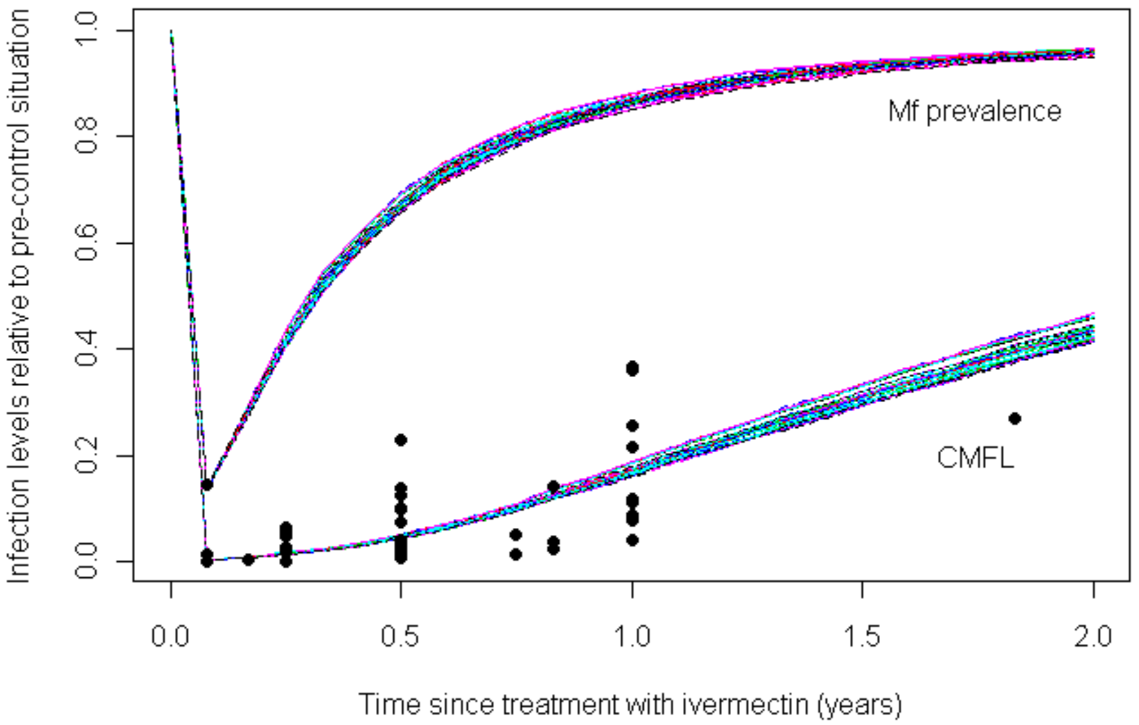

Further, exploratory simulations indicated that the mean longevity of a plug of dead mf was between 2.0 and 4.0 years (given an exponential distribution). By means of a high-resolution grid search (changing the mean duration by a month for each grid step), we determined that the best model fit was obtained with a plug longevity between 40 and 44 months (Figure A2). The data were not informative enough to allow the further pinpointing of the estimate, and therefore we adopted the estimate of 42 months (3.5 years). This estimate is equivalent to assuming that 5% of female worm can release mf again (if inseminated) within 2 months after exposure to ivermectin. Similarly, 25% of female worms can release mf within 1 year, 50% within 2.5 years, 75% within 5 years, and 95% of the female worms can release mf within 10.5 years (an age they do not necessarily reach). In other words, ivermectin was assumed to effectively sterilize a small fraction of the worms, in addition to macrofilaricidal effects.

References

1. Plaisier AP, van Oortmarssen GJ, Habbema JD, Remme J, Alley ES (1990) ONCHOSIM: a model and computer simulation program for the transmission and control of onchocerciasis. *Comput Methods Programs Biomed* **31**: 43–56.

2. Plaisier AP, van Oortmarssen GJ, Remme J, Habbema JD (1991) The reproductive lifespan of *Onchocerca volvulus* in West African savanna. *Acta Trop* **48**: 271–284.

3. Plaisier AP, Alley ES, Boatin BA, Van Oortmarssen GJ, Remme H, et al. (1995) Irreversible effects of ivermectin on adult parasites in onchocerciasis patients in the Onchocerciasis Control Programme in West Africa. *J Infect Dis* **172**: 204–210.

4. Habbema JDF, Oostmarssen GJ, Plaisier AP (1996) The ONCHOSIM model and its use in decision support for river blindness control. In: Isham V, Medley G. *Models for infectious human diseases - their stucture and relation to data*. Cambridge: Cambridge University Press. pp. 360–380.

5. Plaisier AP, Alley ES, van Oortmarssen GJ, Boatin BA, Habbema JD (1997) Required duration of combined annual ivermectin treatment and vector control in the Onchocerciasis Control Programme in west Africa. *Bull World Heal Organ* **75**: 237–245.

6. Winnen M, Plaisier AP, Alley ES, Nagelkerke NJD, van Oortmarssen G, et al. (2002) Can ivermectin mass treatments eliminate onchocerciasis in Africa? *Bull World Heal Organ* **80**: 384–391.

7. Coffeng LE, Stolk WA, Zouré HGM, Veerman JL, Agblewonu KB, et al. (2013) African Programme for Onchocerciasis Control 1995-2015: model-estimated health impact and cost. *PLoS Negl Trop Dis* **7**: e2032.

8. Plaisier AP, van Oortmarssen GJ, Remme J, Alley ES, Habbema JD (1991) The risk and dynamics of onchocerciasis recrudescence after cessation of vector control. *Bull World Heal Organ* **69**: 169–178.

9. United Nations Department of Economic and Social Affairs Population Division (2013) World Population Prospects: The 2012 Revision, Volume I: Comprehensive Tables.

10. Plaisier AP (1996) Modelling onchocerciasis transmission and control.

11. Alley ES, Plaisier AP, Boatin BA, Dadzie KY, Remme J, et al. (1994) The impact of five years of annual ivermectin treatment on skin microfilarial loads in the onchocerciasis focus of Asubende, Ghana. *Trans R Soc Trop Med Hyg* **88**: 581–584.

12. Duke BO (1980) Observations on Onchocerca volvulus in experimentally infected chimpanzees. *Tropenmed Parasitol* **31**: 41–54.

13. Prost A (1980) [Latency period in onchocerciasis]. *Bull World Heal Organ* **58**: 923–925.

14. Albiez EJ (1985) Calcification in adult Onchocerca volvulus. *Tropenmed Parasitol* **36**: 180–181.

15. Karam M, Schulz-Key H, Remme J (1987) Population dynamics of Onchocerca volvulus after 7 to 8 years of vector control in West Africa. *Acta Trop* **44**: 445–457.

16. Schulz-Key H, Karam M (1986) Periodic reproduction of onchocerca volvulus. *Parasitol Today* **2**: 284–286.

17. Schulz-Key H (1990) Observations on the reproductive biology of Onchocerca volvulus. *Acta Leiden* **59**: 27–44.

18. Dadzie KY, Remme J, Rolland A, Thylefors B (1986) The effect of 7-8 years of vector control on the evolution of ocular onchocerciasis in West African savanna. *Trop Med Parasitol* **37**: 263–270.

19. Prost A, Vaugelade J (1981) [Excess mortality among blind persons in the West African savannah zone]. *Bull World Heal Organ* **59**: 773–776.

20. Kirkwood B, Smith P, Marshall T, Prost A (1983) Relationships between mortality, visual acuity and microfilarial load in the area of the Onchocerciasis Control Programme. *Trans R Soc Trop Med Hyg* **77**: 862–868.

21. World Health Organization (1989) Onchocerciasis Control Programme in West Africa: report of the annual OCP research meeting, 20-24 March 1989 (unpublished).

22. Phillipon B (1977) Étude de la transmission d'*Onchocerca volvulus* (Leuckart, 1893) (*Nematoda*, *Onchocercidae*) par *Simulium damnosum* Theobald, 1903 (*Diptera*, *Simuliidae*) en Afrique tropicale Paris: O.R.S.T.O.M.

23. Duke BO (1993) The population dynamics of Onchocerca volvulus in the human host. *Tropenmed Parasitol* **44**: 61–68.

24. Chavasse DC, Post RJ, Davies JB, Whitworth JA (1993) Absence of sperm from the seminal receptacle of female Onchocerca volvulus following multiple doses of ivermectin. *Trop Med Parasitol* **44**: 155–158.

25. Kläger S, Whitworth JA, Post RJ, Chavasse DC, Downham MD (1993) How long do the effects of ivermectin on adult *Onchocerca volvulus* persist? *Trop Med Parasitol* **44**: 305–310.

26. Cupp EW, Duke BO, Mackenzie CD, Guzmán JR, Vieira JC, et al. (2004) The effects of long-term community level treatment with ivermectin (Mectizan) on adult *Onchocerca volvulus* in Latin America. *Am J Trop Med Hyg* **71**: 602–607.

27. Cupp EW, Cupp MS (2005) Short report: impact of ivermectin community-level treatments on elimination of adult *Onchocerca volvulus* when individuals receive multiple treatments per year. *Am J Trop Med Hyg* **73**: 1159–1161.

28. Duke BO, Pacqué MC, Muñoz B, Greene BM, Taylor HR (1991) Viability of adult *Onchocerca volvulus* after six 2-weekly doses of ivermectin. *Bull World Heal Organ* **69**: 163–168.

29. Duke BO, Zea-Flores G, Castro J, Cupp EW, Muñoz B (1990) Effects of multiple monthly doses of ivermectin on adult *Onchocerca volvulus*. *Am J Trop Med Hyg* **43**: 657–664.

30. Duke BO, Zea-Flores G, Castro J, Cupp EW, Muñoz B (1991) Comparison of the effects of a single dose and of four six-monthly doses of ivermectin on adult *Onchocerca volvulus*. *Am J Trop Med Hyg* **45**: 132–137.

31. Duke BO, Zea-Flores G, Castro J, Cupp EW, Muñoz B (1992) Effects of three-month doses of ivermectin on adult *Onchocerca volvulus*. *Am J Trop Med Hyg* **46**: 189–194.

32. Duke BOL (2005) Evidence for macrofilaricidal activity of ivermectin against female *Onchocerca volvulus*: further analysis of a clinical trial in the Republic of Cameroon indicating two distinct killing mechanisms. *Parasitology* **130**: 447–453.

33. Gardon J, Boussinesq M, Kamgno J, Gardon-Wendel N, Demanga-Ngangue, et al. (2002) Effects of standard and high doses of ivermectin on adult worms of *Onchocerca volvulus*: a randomised controlled trial. *Lancet* **360**: 203–210.

34. Kläger SL, Whitworth JA, Downham MD (1996) Viability and fertility of adult *Onchocerca volvulus* after 6 years of treatment with ivermectin. *Trop Med Int Heal* **1**: 581–589.

35. Basáñez M-G, Pion SDS, Boakes E, Filipe JAN, Churcher TS, et al. (2008) Effect of single-dose ivermectin on *Onchocerca volvulus*: a systematic review and meta-analysis. *Lancet Infect Dis* **8**: 310–322.
